# Tailored NEOadjuvant epirubicin, cyclophosphamide and Nanoparticle Albumin-Bound paclitaxel for breast cancer: The phase II NEONAB trial—Clinical outcomes and molecular determinants of response

**DOI:** 10.1371/journal.pone.0210891

**Published:** 2019-02-14

**Authors:** Caitlin Murphy, Andrea Muscat, David Ashley, Violet Mukaro, Linda West, Yang Liao, David Chisanga, Wei Shi, Ian Collins, Sally Baron-Hay, Sujata Patil, Geoffrey Lindeman, Mustafa Khasraw

**Affiliations:** 1 University Hospital Geelong, Geelong, Victoria, Australia; 2 School of Medicine, Deakin University, Geelong, Victoria, Australia; 3 Preston Robert Tisch Brain Tumor Center, Duke University, Durham, North Carolina, United States of America; 4 Lake Imaging, Geelong, Victoria, Australia; 5 The Walter and Eliza Hall Institute of Medical Research, Parkville, Victoria, Australia; 6 South West Health Care, Warrnambool, Victoria, Australia; 7 Royal North Shore Hospital, St Leonards, New South Wales, Australia; 8 North Shore Private Hospital, St Leonards, New South Wales, Australia; 9 Memorial Sloan Kettering Cancer Center, New York, United States of America; 10 National Health and Medical Research Council Clinical Trials Centre, University of Sydney, New South Wales, Australia; Istituto di Ricovero e Cura a Carattere Scientifico Centro di Riferimento Oncologico della Basilicata, ITALY

## Abstract

**Background:**

This study evaluated the feasibility of achieving high response rates in stage II or III breast cancer by tailoring neoadjuvant therapy using clinical and histopathological features and the Oncotype DX Breast Recurrence Score. Genomic determinants of response and resistance were also explored.

**Patients and outcome measures:**

Fifty-one patients were enrolled. The primary cohort comprised 40 patients: 15 human epidermal growth factor receptor type 2 (HER2)-amplified; 15 triple-negative (TNBC); and ten hormone receptor (HR)-positive, HER2-non-amplified tumours; with recurrence scores ≥25. Patients were treated with epirubicin and cyclophosphamide, followed by nab-paclitaxel, with the addition of trastuzumab if HER2-amplified. The primary endpoint was pathological complete response (pCR) in the breast. Pre- and post-treatment tumour samples underwent variant burden, gene and gene pathway, mutational signature profile and clonal evolution analyses.

**Results:**

The pCR rates were: overall 55% (*n* = 22), HER2-amplified 80% (*n* = 12), triple-negative 46% (*n* = 7) and HR-positive, HER2-non-amplified 30% (*n* = 3). Grade 3 or 4 adverse events included febrile neutropenia (8%), neutropenia (18%), sensory neuropathy (5%), deranged transaminases (5%), fatigue (2%), diarrhoea (2%), and pneumothorax (2%). Molecular analyses demonstrated strong similarities between residual disease and matched primary tumour. ATM signalling pathway alterations and the presence of a COSMIC Signature 3 implied the majority of tumours contained some form of homologous repair deficiency. ATM pathway alterations were identified in the subset of TNBC patients who did not achieve pCR; Signature 3 was present in both pCR and non-pCR subgroups. Clonal evolution analyses demonstrated both persistence and emergence of chemoresistant clones.

**Conclusions:**

This treatment regime resulted in a high rate of pCR, demonstrating that tailored neoadjuvant therapy using a genomic recurrence score is feasible and warrants further investigation. Molecular analysis revealed few commonalities between patients. For TNBC future clinical gains will require precision medicine, potentially using DNA sequencing to identify specific targets for individuals with resistant disease.

**Trial registration:**

Clinicaltrials.gov NCT01830244

## Introduction

In early breast cancer, gene expression profiles in hormone receptor (HR) positive disease and human epidermal growth factor type 2 receptor (HER2) status define the benefit a patient is likely to receive from systemic therapy and are therefore used to guide treatment [1]. For triple-negative breast cancer (TNBC), except for the small minority of *BRCA* germline mutation carriers, no such predictors exist [2]. Both inter- and intra-tumoural heterogeneity pose significant barriers to the development of predictive markers and targeted therapies [3]. Efforts to enhance our molecular understanding of these cancers are necessary to improve outcomes for patients with this disease.

Advances in high-throughput sequencing have allowed the genomic landscape of primary breast cancer to be described in extraordinary detail [4–6]. The molecular profile of residual disease post-neoadjuvant therapy is comparatively incomplete. Early studies of neoadjuvant therapies which included the assessment of residual disease have focused on gene expression changes or were limited to targeted panels of fewer than 200 genes [7–9]. More recent reports utilising whole exome or whole genome sequencing have increased our understanding, but the number of patients and tumours studied remains relatively small [10–12].

Although controversy still exists over the use of pathological complete response (pCR) as a surrogate endpoint in breast cancer trials, patients who achieve a pCR in general, and particularly in the TNBC subgroup, have better prognosis [13, 14]. Neoadjuvant studies provide an opportunity to examine the evolution of resistant cancers under the selective pressure of chemotherapy and determine how their genomic profiles differ from those of complete responders.

Here we present the clinical outcomes of the NEONAB trial and describe genomic alterations present in some of the TNBC subset, comparing those that achieved a pCR after treatment with anthracycline/taxane chemotherapy to those with residual disease. By identifying alterations that emerged in residual disease we aimed to study how these individual cancers evolved from their primary tumours.

Our analysis of gene pathways and mutational signatures revealed evidence of homologous repair deficiency (HRD) in most tumour samples. Clonal evolution analysis demonstrated the persistence and emergence of chemoresistant clones between diagnostic and residual disease. There were few commonalities identified amongst the TNBCs included in our cohort, underscoring the known genomic heterogeneity of this disease and the need for a personalised medicine approach to the treatment of chemorefractory patients.

## Materials and methods

### Study design

NEONAB was a multicentre open-label phase II study (Clinicaltrials.gov #: NCT01830244) conducted at three Australian centres. The primary objective was to measure pCR rate in the breast. Secondary objectives included pCR in breast and axillary lymph nodes (LN), pCR and near-complete response (nCR) combined, disease-free survival (DFS), rate of breast-conserving surgery, tolerability, and safety of the investigated regimens. This study was reviewed and approved by the relevant institutional ethics committees at each recruitment site (University Hospital, Geelong; South West Health Care, Warrnambool; Royal North Shore Hospital, Sydney). All patients signed written informed consent forms.

### Patient eligibility

Patients with previously untreated stage II or III, unilateral histologically confirmed invasive breast cancer and an Eastern Cooperative Oncology Group (ECOG) performance status ≤ 1 were eligible. All tumours were tested locally for oestrogen receptor (ER) and progesterone receptor (PR) status, and were considered positive if ≥1% of tumour cells stained for ER and/or PR. HER2 status was assessed by immunohistochemistry and in situ hybridisation. Other inclusion criteria were age ≥ 18 years, normal left ventricular ejection fraction, and adequate haematopoietic, liver and renal function. Germline testing for *BRCA* status was not required or undertaken.

Exclusion criteria included distant metastases, history of ipsilateral breast cancer, previous radiation therapy to the breast, previous anthracyclines or taxanes, serious cardiovascular, hepatic, neurologic or renal comorbid conditions, pregnancy or lactation, and male sex.

### Treatment

Patients with HER2-non-amplified, HR-positive cancers had the recurrence score (RS) assay performed according to screening requirements. The primary cohort consisted of patients with HER2-non-amplified, HR-positive, RS ≥25 tumours, HER2-amplified tumours and TNBCs. They received epirubicin 90 mg/m^2^ and cyclophosphamide 600 mg/m^2^ (EC) every three weeks for 12 weeks; followed by nab-paclitaxel 125 mg/m^2^ on days 1, 8, and 15 every four weeks for 12 weeks. Trastuzumab was added to nab-paclitaxel for HER2-amplified patients at an initial dose of 8 mg/kg and subsequent doses at 6 mg/kg every 21 days for a total of 12 months.

Grade ≥3 non-haematologic toxicities mandated a 20% dose reduction. Nab-paclitaxel was withheld for grade ≥2 neuropathy until resolved to ≤ grade 1. Trastuzumab was continued if chemotherapy was delayed.

Patients underwent mastectomy or breast-conserving surgery, and sentinel node biopsy or axillary dissection after discussion with their surgeon. Postoperative radiotherapy and adjuvant endocrine therapy for HR-positive patients were determined by a breast cancer tumour board/multidisciplinary meeting.

### Clinical endpoint assessment

Residual tumour was evaluated microscopically by local pathologists according to predefined criteria. pCR in the breast was defined as ypT0/is ypN0–3, no evidence of invasive tumour cells in the surgical breast specimen, with residual ductal carcinoma in situ permitted. Invasive or non-invasive disease in LN was allowed. nCR was defined as the presence of scattered tumour cells only [15]. DFS was defined as the time from registration on the trial until documented evidence of breast cancer recurrence or death from breast cancer. Primary tumour progression during neoadjuvant treatment was not considered an event. Complete blood count and metabolic profiles were assessed at every treatment cycle. All dose reductions, delays, and adverse events (AE) were reported. The Common Toxicity Criteria for Adverse Events (CTCAE), Version 4.0, was used.

### Statistical methods

In previous studies of neoadjuvant therapy, pCR have ranged from 12 to 30% [13, 15, 16]. Accordingly, the RR for the null hypothesis (uninteresting rate) was set at 30% and for the alternative (worthy of further study) at 50%. A single-stage binomial design was planned to discriminate between overall pCR rates of 30% and 50% with a type I error of 6% and 87% power. This required a total of 40 patients, and if ≥ 17 patients were to have pCR, the regimen was to be deemed worthy of further study. A maximum of 15 patients with HER2-amplified tumours or TNBC were allowed.

The evaluable intention-to-treat population included all patients enrolled with HER2-amplified, TNBC, and eligible HR-positive breast cancers who received at least one cycle of neoadjuvant chemotherapy and at least one post-infusion tumour assessment. DFS was evaluated using Kaplan-Meier methods.

### Tumour samples

Patients enrolled on the NEONAB study consented to the use of their tumour tissue and blood for translational research. Formalin fixed paraffin embedded (FFPE) pre-treatment core biopsies of breast lesions and involved LN were obtained for diagnostic purposes and 20 paraffin scrolls of 10 micron thickness were provided for DNA extraction. For those patients who had an incomplete pathological response, FFPE samples of the residual disease were taken from the surgical specimen. Whole blood samples were available for germline DNA extraction.

### DNA extraction

Genomic DNA was extracted from FFPE TNBC tumours using the ReliaPrep FFPE gDNA Miniprep System (Promega) according to the manufacturer’s protocol. The DNA was quantified using a Qubit dsDNA HS Assay kit and a Qubit 2.0 Fluorometer (ThermoFisher).

### Library preparation and whole exome sequencing

Libraries were prepared starting with 200ng DNA. For FFPE derived DNA, a pre-capture library was prepared using the Agilent SureSelect XT Target Enrichment System with SureSelect XT-Adaptor Libraries (Version 2.5B). This protocol uses the KAPA Hyper Prep Kit (KAPA Biosystems, Version KR0961 –v1.14). DNA was purified using 1.8x AMPureXP beads. Libraries prepared from germline DNA derived from blood and all capture and post-capture steps were performed using the Agilent SureSelectXT Target Enrichment System according to protocol G7530-90000, Version C0, December 2016. Whole exome sequencing was performed using the Illumina HiSeq 3000 system, Protocol 15066493. Four libraries per lane were pooled to achieve coverages of 150x for the diagnostic and residual disease samples. For the germline samples, libraries were pooled in a single lane to achieve coverages of 50x. Library preparation and sequencing was conducted by the Monash Health Translational Precinct (MHTP) Medical Genomics Facility (Melbourne, Australia).

### Data processing and variant calling

Paired-end reads generated from 27 samples (16 tumour samples and 11 germline samples) were mapped to the GRCh38/hg38 genome using the Subread aligner (Version 1.5.3) [17]. Only uniquely mapped fragments were reported, and short insertions/deletions (INDELs) up to 16 base pairs (bp) in length were detected during read mapping. Single nucleotide variants (SNVs) were then called from the aligned reads using exactSNP [18]. Low quality bases in reads were removed and each read was also trimmed by three bases from each end. A q-value cutoff of 12 was applied for SNV calling. When calling SNVs in a tumour sample, each SNV was also required to have a minimum sequencing depth of 50x in this sample and in the matched germline sample this SNV had to have at least 20x sequencing depth and its mismatch percentage had to be lower than 10%. Further filtering excluded SNVs with a variant allele frequency (VAF) less than 10%. It was accepted that some rare, low frequency variants may be excluded from the analysis due to the stringency of these parameters.

Variants were annotated using Variant Effect Predictor [19] and non-synonymous SNVs (missense, stop-gained, splice site) were selected for gene and gene pathway analyses. For the gene and gene pathway analyses, variants assigned as intronic or intergenic were discarded. Variants were then categorised as high functional impact (HFI) variants if deemed likely to be deleterious or damaging by at least three of six functional prediction tools (SIFT, PolyPhen, PROVEAN, Mutation Taster, Mutation Assessor, LRT score) [19].

INDELs were called using the Subread aligner [17] and the results were filtered by five conditions. An INDEL must have at least 20 supporting reads in the sample where it was detected and at least five supporting reads where the INDEL is at least 25 bp away from both ends of the read. An INDEL must have at least 20 reads covering the same chromosomal location but have no INDELs at the same location in the matched germline sample. The matched germline sample could not contain any INDELs within a 20-bp region surrounding an INDEL detected in the tumour sample. Finally, an INDEL must have had a mismatch rate less than 10% in the surrounding 10-bp region in the sample where it was detected. Those with VAF less than 10% or annotated as intergenic or intronic were excluded. To minimise false positive calls INDELs adjacent to more than five repetitive regions were excluded. All INDELs were considered HFI.

### Variant validation

To exclude sequencing artefacts, where DNA was available HFI variants with a VAF greater than 20% were validated by Sanger sequencing. Primers were designed using NCBI Primer-Blast [20], Integrated DNA Technologies OligoAnalyser tool [21] and UCSC In-Silico PCR tool [22] (S1 Table). Primers were tested and cycling conditions optimised prior to use on tumour samples. Tumour DNA underwent 35 cycles of PCR amplification and adequate product was confirmed using gel electrophoresis prior to sequencing. Variants were manually reviewed using Chromas Lite v2.01 and BioEdit Sequence Alignment Editor v7.0.9.0 software packages.

### Sequencing metrics

The number of bases deemed callable was determined using the GATK CallableLoci [23] tool with a minimum depth set at 20 reads. Mutation burden was calculated as total number of SNVs, including synonymous, intronic, intergenic variants and INDELs per Mb for each sample. A second analysis looking only at non-synonymous SNVs, splice site variants and INDELs was performed.

### Gene pathway analysis

Gene pathway analyses were conducted to assess the relationship between any genes that possessed HFI SNVs and INDELs. For each sample, gene lists were used as input in DAVID [24, 25] and analysed using the functional annotator. Enrichment in Biocarta and Kegg pathways was assessed using a modified Fisher’s Exact test. The null hypothesis was rejected at the 0.05 significance level. Significantly enriched pathways were manually reviewed in each sample and comparisons were made between diagnostic samples that obtained pCR and those that did not and between diagnostic and paired residual disease samples. Gene pathways are illustrated using cBioportal Oncoprint tool [26].

### Mutational signature analysis

Variants included in the mutational signature analysis had a VAF >10% and quality score > 20. Non-synonymous, intronic, intergenic and synonymous SNVs were all included in this analysis. Each was sample was individually analysed using DeconstructSignatures [27] with default normalisation and a minimum signature contribution of 0.06. Signatures detected in the NEONAB samples were referenced against the 30 signatures found in the latest COSMIC classification [28].

### Clonal evolution analysis

Paired-end reads from 11 patients were first aligned to the human reference genome HG38 using BWA-MEM [29] with default parameters. After alignment, the BAM files were processed according to GATK best practices [30] followed by calling of consensus variants using VarScan [31] mpileup2cns function with p-value, strand-filter and minimum variant filter set to 0.05, 0 and 0.05 respectively. To identify clonal sub-populations in each patient, clonal analysis was performed using superFreq [32], a cancer exome clonality inference tool. Of the 11 patients, five had germline, diagnostic and residual disease samples available for analysis. These five patients’ samples were analysed using the germline data from the six patients who achieved pCR as controls. superFreq was run using default parameters according to the manual’s protocol. Clonal analysis was performed independently for each patient across the different sample condition types.

## Results

### Patient characteristics

**Fig 1:**
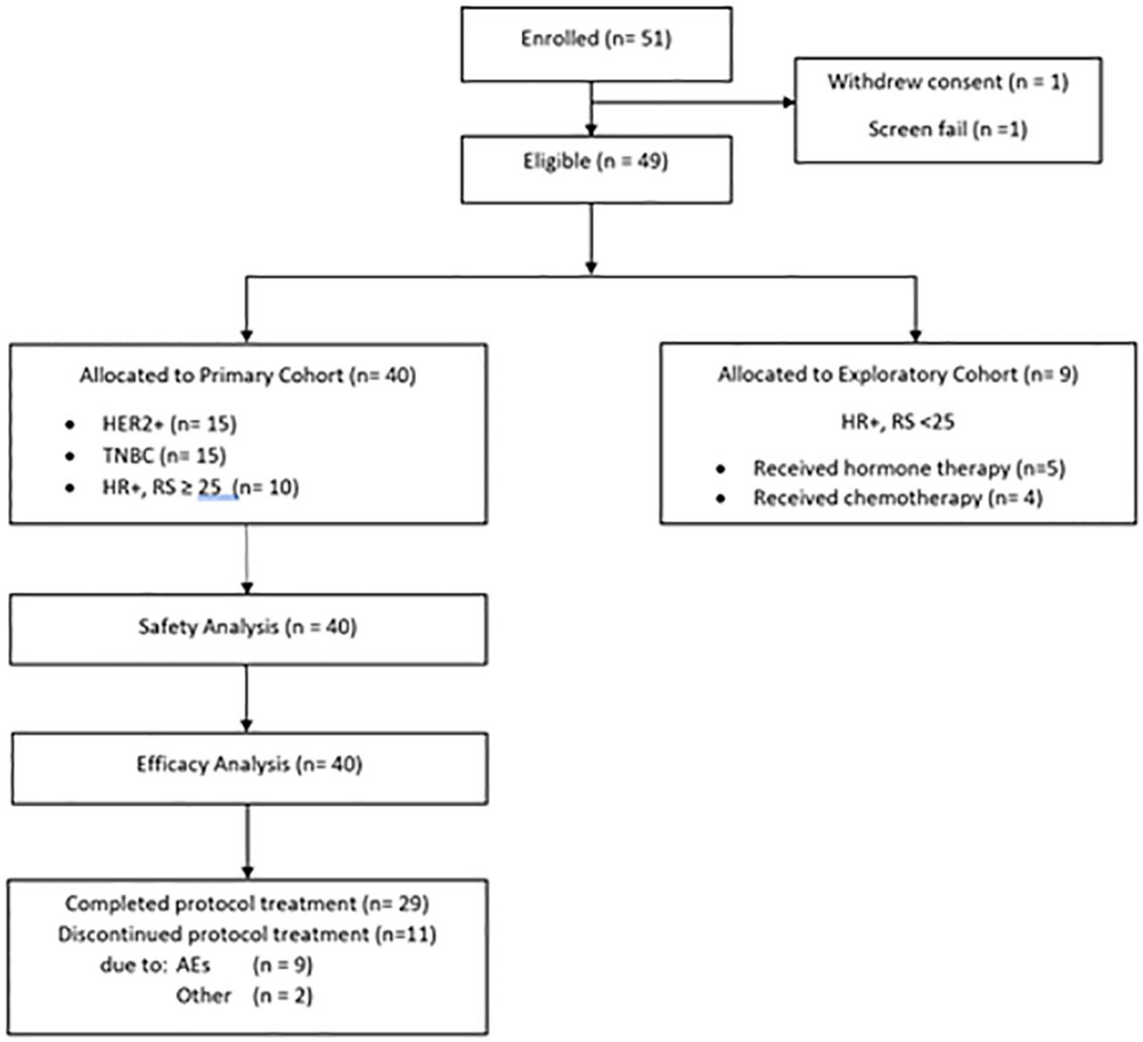
CONSORT flowchart.

Between April 2013 and December 2015, 51 patients were enrolled. At 31 December 2015, the median follow-up was 18.6 months (range 11.52–31.56 months). The primary cohort consisted of 40 women (15 HER2-amplified, 15 TNBC, and ten HER2-non-amplified, HR-positive patients with RS ≥ 25). Baseline characteristics are summarised in Table 1. A further nine women were in a separate exploratory cohort of neoadjuvant hormonal treatment or a short course of chemotherapy at the discretion of the treating physician (Fig 1). Two patients were treated off study, one withdrew consent, and one was found to be ineligible after enrolment. All 51 were included in the analysis of the response rates, but only those with TNBC are described in terms of genomic analysis.

**Table 1:** Baseline characteristics of patients in the NEONAB primary cohort.

Characteristic	n	%
Age, years		
Median	51	
Range	(35–77)	
ECOG		
0	39	98
1	1	2
Ethnic group		
Caucasian	37	93
Asian	3	8
Tumour pathology		
Adenocarcinoma	1	2
Ductal no special type	34	85
Inflammatory	1	2
Lobular carcinoma	1	2
Mucinous carcinoma	1	2
Not specified	2	5
Oestrogen receptor status		
Negative*	22	55
Positive	18	45
Progesterone receptor status		
Negative*	27	68
Positive	13	32
HER2 status		
Non-amplified	25	63
Amplified	15	37
Tumour grade		
2	14	35
3	22	55
Not specified	4	10
Tumour stage+		
2a	10	25
2b	12	30
3a	14	35
3b	3	8
3c	1	2

*HR status deemed negative if <1% nuclei staining; + American Joint Committee on Cancer Breast Cancer Staging v.7; ECOG, Eastern Cooperative Oncology Group; HER2, human epidermal growth factor receptor type 2

Locally assessed high Ki67 (Ki67≥15) did not correlate with a high RS result (RS≥25) in five of the 17 (30%) of the patients who had both results available.

### Treatment response

In the primary cohort, the overall pCR rate in the breast (Table 2) was 55% (*n =* 22). Of these 32.5% (*n =* 13) were ypT0 ypN0, 12.5% (*n =* 5) ypT0/is ypN0, and 10% (*n =* 4) were ypT0/is ypN+. The pCR rate in the breast alone varied according to subtype: HER2-amplified, 80% (*n =* 12), TNBC 46% (*n =* 7) and HR-positive, HER2-non-amplified 30% (*n =* 3). An additional 10% (*n =* 4) achieved nCR, defined as residual scattered tumour cells only. The combined rate of pCR and nCR in the breast only was therefore 65% (*n =* 26). This compares favourably with historical controls [33].

**Table 2:** Summary of pathological complete response rates in different subsets.

Site	All (*n* = 40)	HER2+ (*n* = 15)	TNBC (*n* = 15)	HR+ HER2- RS ≥ 25* (*n* = 10)
Breast only	55(22)	80 (12)	46 (7)	30(3)
Breast and LN	45(18)	80 (12)	40 (5)	10(1)

*HR+ HER2- RS ≥ 25: HR-positive, HER2-non-amplified with recurrence score ≥ 25 HER2+: human epidermal growth factor receptor type 2 amplified; TNBC: triple-negative breast cancer; HR: hormone receptor

The secondary endpoint, pCR rate in breast and LN, was 45%, with similar distributions according to subtype (Table 2).

### Surgery and disease-free survival

**Fig 2:**
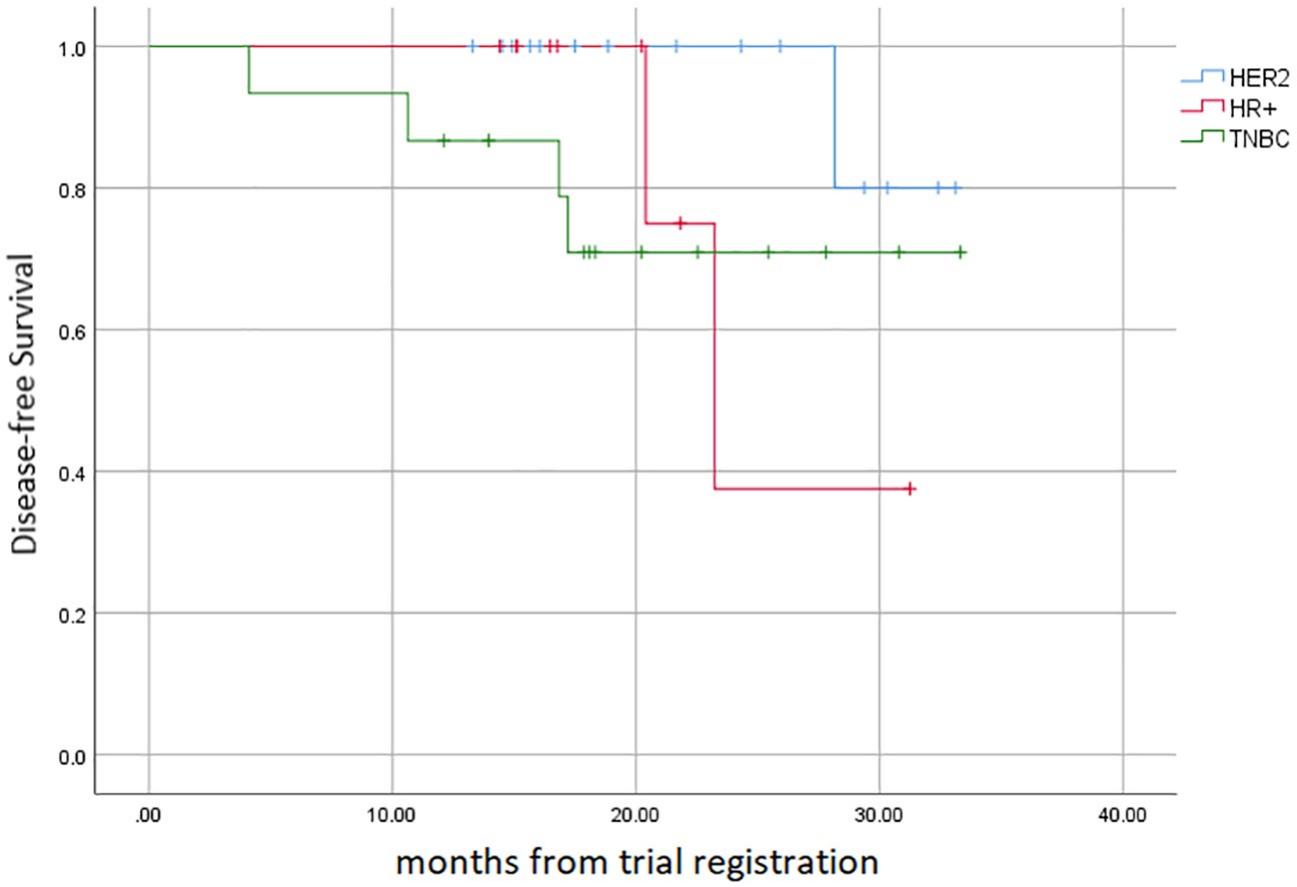
Kaplan-Meier estimates for DFS according to breast cancer subtype.

The breast conservation rate was 47.5% (*n =* 19). At a median of 18 months of follow-up, disease had recurred in seven patients; four TNBC, one HER2-amplified and two HR-positive patients. Early DFS data for the entire cohort are presented in Fig 2. The median DFS has not yet been reached. Five of these patients had residual disease after protocol therapy and two had achieved pCR defined as ypT0/is.

### Tolerability and safety

Overall, treatment was well tolerated, with 97.5% (*n =* 39) completing protocol therapy of EC, and 72.5% (*n =* 29) patients completing protocol nab-paclitaxel. Most of those who did not complete nine doses of nab-paclitaxel according to the protocol required minor dose reductions or omissions due to AEs, most commonly neuropathy. Overall, the relative dose intensity for nab-paclitaxel was 73%, with 88% of the cohort (*n =* 35) receiving ≥85% of the scheduled dose. Common AEs are summarised in Table 3. G-CSF was given to 77.5% (*n =* 31) of patients during EC and 62.5% of patients during nab-paclitaxel (*n =* 25) on days 2–7 during weekly therapy. The rate of sensory neuropathy with nab-paclitaxel was 55% (*n =* 22), with most events ≤ grade 1. Grade 3 sensory neuropathy occurred in 5% (*n =* 2). Of the two cases of grade 3 transaminase derangement, only one was suspected to be related to nab-paclitaxel and both patients recovered to ≤ grade 1.

**Table 3:** Most common adverse events*.

Adverse event	Any grade		Grade 3 or 4	
	*N*	%	*n*	%
Alopecia	38	95	-	-
Anxiety	6	15	-	-
Bone pain	6	15	-	-
Constipation	9	23	-	-
Deranged transaminases	2	5	2	5
Diarrhoea	8	20	1	2
Dysgeusia	6	15	-	-
Dyspepsia	4	10	-	-
Fatigue	27	68	1	2
Febrile neutropenia	3	8	3	8
Gastro-oesophageal reflux	8	20	-	-
Headache	6	15	-	-
Insomnia	7	18	-	-
Nausea	25	63	-	-
Neutropenia	23	58	7	18
Sensory neuropathy	22	55	2	5
Pneumothorax	1	2	1	2

* Any grade reported for at least 10% of patients or grade ≥3 using CTCAE Version 4.0 reported for any patient. If a patient had more than one adverse event within a preferred term, the patient was counted once in the term.

### Tumour samples

There were 40 patients included in the NEONAB primary cohort. Three did not consent to the use of their tissue and two others withdrew consent. Tissue could not be obtained for three patients, and two patients had multifocal disease with two diagnostic samples per patient available. In total, 34 diagnostic and 19 residual disease samples were obtained for analysis. There were 15 TNBC diagnostic and eight residual disease specimens. These samples underwent DNA extraction. Adequate DNA (minimum 200 ng) was extracted from 11 of the 15 diagnostic and five of eight residual disease samples, and these proceeded to library preparation and whole exome sequencing. Of the six patients who achieved pCR, none developed breast cancer recurrence or died, whereas three of the five patients with residual disease have recurred and two of these patients have succumbed to their disease.

### Data processing, variant calling and variant validation

Sequencing quality was above specification. There was an average of 10 000 somatic SNVs per diagnostic sample and an average of 13 500 SNVs per residual disease sample. After filtering for coverage, base quality, and minimum VAF, diagnostic samples contained an average of 203 SNVs (range 40–599) and residual disease samples contained an average of 143 SNVs (range 22–310). Annotation to determine functional impact yielded an average of 30 HFI SNVs (range 5–101) per diagnostic sample and an average of 17 HFI SNVs (range 1–49) per residual disease sample. Stringent filtering removed the majority of INDELs, leaving an average of 88 INDELs (range 49–200) called from the diagnostic samples and an average of 80 INDELs (range 39–114) from residual disease. There was adequate DNA for variant validation in ten of the 16 tumour samples. Ninety-four percent of SNVs (33/35) and 72% of INDELs (5/7) were validated using Sanger sequencing.

### Variant burden

The total number of variants ranged from 1.0–9.3 variants/Mb in the diagnostic samples and 0.70–4.6/Mb in the residual samples. Analysis of the number of non-synonymous variants was similar; 0.72–4.6/Mb in the diagnostic and 0.60–2.3/Mb in the residual tumours. Two patients exhibited an increase in variant load (N07, N08), another two remained stable (N09, N27) and one showed a reduction post-treatment (N29). There was also no difference in variant burden between the group that achieved pCR and the non-pCR group, the mean number of alterations were 3.7/Mb vs 2.9/Mb, respectively. The results were similar when the analysis was restricted to non-synonymous variants only, with 1.9 variants/Mb for the pCR group vs 1.5 variants/Mb for the non-pCR group.

### Alterations in TP53 and SWitch/Sucrose Non-Fermentable (SWI/SNF) complex genes

Consistent with the known heterogeneity of TNBC, most of the functional mutations identified in our samples were not recurrent. Alterations in *TP53* were found in eight of 16 tumour samples, six diagnostic and two residual disease. The type of alteration in *TP53* varied from patient to patient and included both INDELs and missense SNVs. The same *TP53* variants found in D07 and D27 were also found in their paired residual disease samples, R07 and R27. There was no association between *TP53* alterations and chemotherapy response. Truncating mutations in *ARID1A* and *ARID1B*, genes encoding subunits of the SWI/SNF chromatin regulating complex, were identified in two residual disease samples, R09 and R07, which were not present in the matched diagnostic samples. Alterations in *ARID1A* were also observed in D20 and D21, samples from patients that did achieve pCR.

### Gene pathway comparisons and ATM signalling in chemoresistant tumours

**Fig 3:**
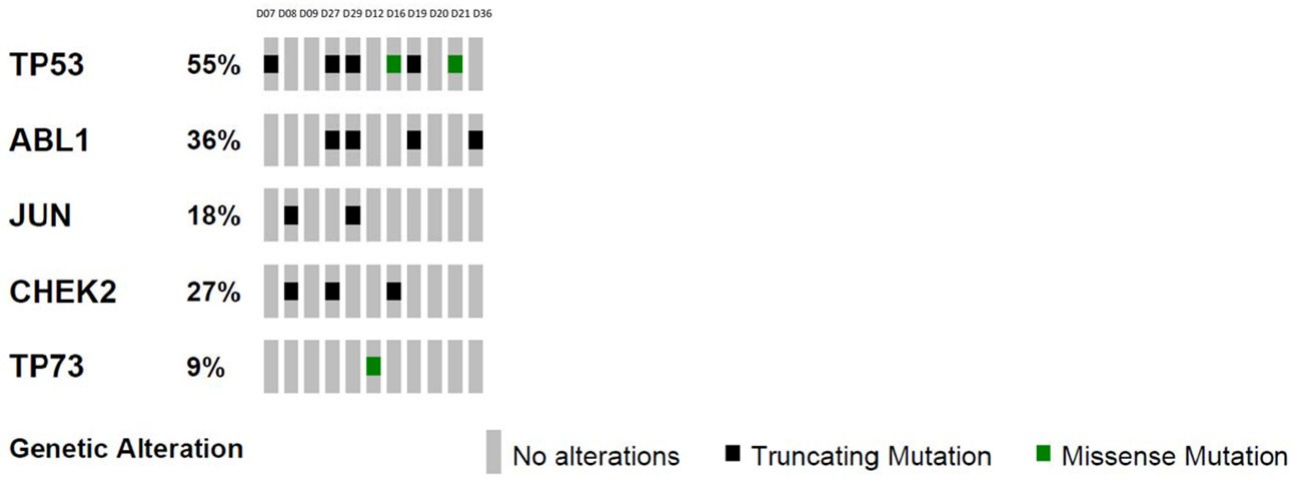
Functional mutations in the ATM signalling pathway. OncoPrint of variants detected in five genes central to the ATM signalling pathway. The percentage samples affected by variants in each gene are noted and missense (green) and truncating (black) mutations are presented.

Three of the five diagnostic samples (D08, D27, D29) from the non-pCR group were found to be significantly enriched for functional mutations in the Biocarta ATM signalling pathway (*p* < 0.05; Fig 3). Manual review of germline sequencing data for these patients revealed no HFI variants or INDELs in BRCA1, BRCA2 or ATM. Although alterations in this pathway were also identified in sample D07 and in five of six diagnostic samples from the pCR group, these did not reach statistical significance (*p* > 0.05). None of the paired residual disease samples retained this enrichment. Analysis of the diagnostic samples from the group that achieved pCR revealed several significantly enriched pathways, but these varied from sample to sample, with no commonalities identified (S2 Table). Similarly, analysis of the non-pCR group did not reveal any significantly enriched pathways shared across multiple samples. The small sample size precludes definitive comparisons between groups but the lack of commonalities seen between samples from different patients is consistent with the known genomic heterogeneity of this disease.

### Androgen signalling/*FOXA1* pathway alterations

Samples were examined specifically for alterations in the androgen receptor-signalling/*FOXA1* pathway. Patients whose tumours contained a single alteration in this pathway were previously reported to have a significantly higher likelihood of achieving pCR to neoadjuvant anthracycline/taxane chemotherapy in TNBC [12]. In our study, the diagnostic samples from two patients who achieved pCR contained a functional mutation in this pathway, but the same alterations were also found in three patients with post-treatment residual disease. Alterations in this pathway were not predictive of response to chemotherapy in our cohort, but this conclusion is limited by the small number of patients assessed.

### Mutational signatures

Mutational base substitution signatures were identified in each diagnostic and residual disease sample. Usually seen in approximately 20% of breast cancers [34], Signature 3 in the COSMIC classification was identified in 75% (12/16) of samples, nine diagnostic and three residual disease. This signature is associated with HRD. Initially identified in samples with germline and somatic *BRCA1/2* variants, more recently it has also been found in tumours with epigenetic silencing of *BRCA1* or homologous deletions of *BRCA1/2* [35]. None of the samples enriched for Signature 3 contained somatic *BRCA1/2* variants in our cohort, suggesting other alterations contributed to enrichment of this signature. In a cohort of 992 breast cancers, Polak et al. [35] demonstrated the majority of tumours highly enriched for Signature 3 did not carry inactivating *BRCA1/2* variants but identified *PALB2* variants and inactivation of *RAD51C* as alternative lesions associated with this signature’s activity. Variants in other genes associated with HRD (Table 4), specifically *CHEK2* and *PTEN*, were identified in five of our 12 samples with Signature 3 activity. In our cohort Signature 3 was equally likely to be identified in diagnostic samples that achieved pCR as those that did not, suggesting no association with response.

**Table 4:** Genes associated with HRD or MMR.

Genes associated with HRD	Genes associated with MMR
*BRCA1*	*MLH1*
*BRCA2*	*MLH3*
*PALB2*	*MSH2*
*CHEK2*	*MSH3*
*RAD51D*	*MSH6*
*RAD51C*	*PMS1*
*EMSY*	*PMS2*
*PTEN*	*POLH*
*BRIP1*	
*ATM*	
*ATR*	
*FAM175A*	
*BARD1*	
*NBN*	
*FANCA*	
*FANCM*	
*RAD50*	

**Fig 4:**
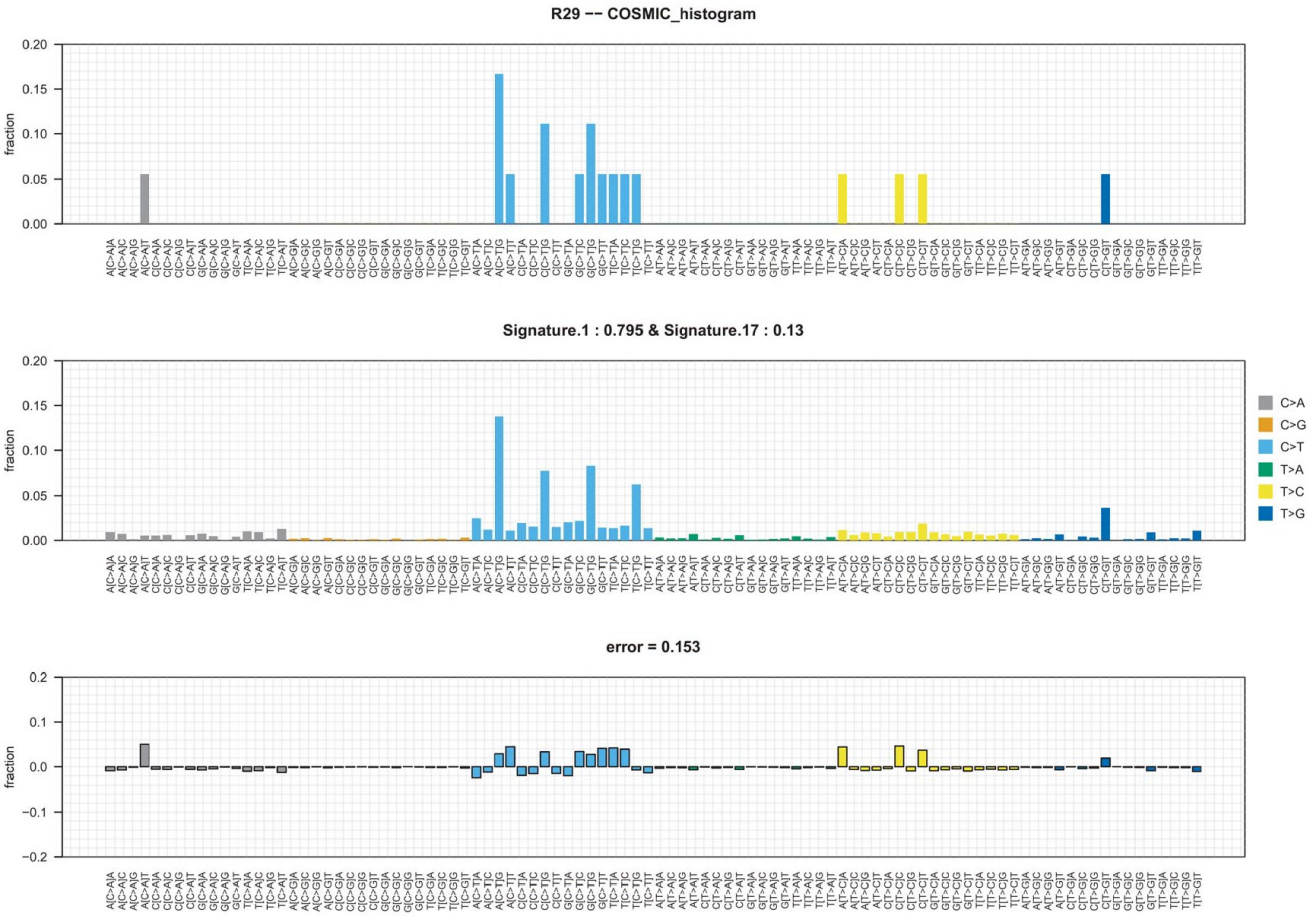
Mutational signature analysis. Signature 17 is highlighted in sample R29. Signature 1 is age-related and is the predominant signature in this sample. Signature 17 contributes to ~13% of the mutation burden.

Signature 17 was identified in the residual disease of one patient, N29 (Fig 4). The aetiology of this signature is unknown, it is more dominant later in breast cancer tumourigenesis [36] and has previously been described as arising in multiple treatment-resistant metastatic lesions in one case of TNBC resistant to both chemotherapy and a *PIK3CA* inhibitor [37]. Of note, both the diagnostic and residual samples from patient N29 harboured a HFI SNV in *PIK3CA*. Patient N29 in our study had only a partial pathological response to chemotherapy yet has remained recurrence-free three years post-surgery. Signature 17 may derive from cells capable of surviving under the selective pressure of, in our case anthracycline/taxane chemotherapy and in the previous case a pan-*PIK3CA* inhibitor, and thus be a marker of treatment insensitivity.

Signatures 6, 20 and 26, associated with mismatch repair (MMR) and microsatellite instability were found collectively in eight of 16 tumours. Of note, only one of these samples, D12, contained variants in the known MMR genes (Table 4) and a higher than average variant burden, at 9.2 variants/Mb. These signatures were present in both groups, those that attained pCR and those that did not, and do not appear to have any relationship to response to chemotherapy.

### Clonal analysis demonstrates persistence and emergence of chemoresistant subclones

Clonal evolution analyses confirmed populations evident in residual disease samples descended from subclones present in their matched primary cancers. This is consistent with previous reports evaluating primary and metastatic disease that suggest the emergence of resistant cells occurs through treatment-induced clonal selection rather than chemotherapy-induced mutagenesis [11].

**Fig 5:**
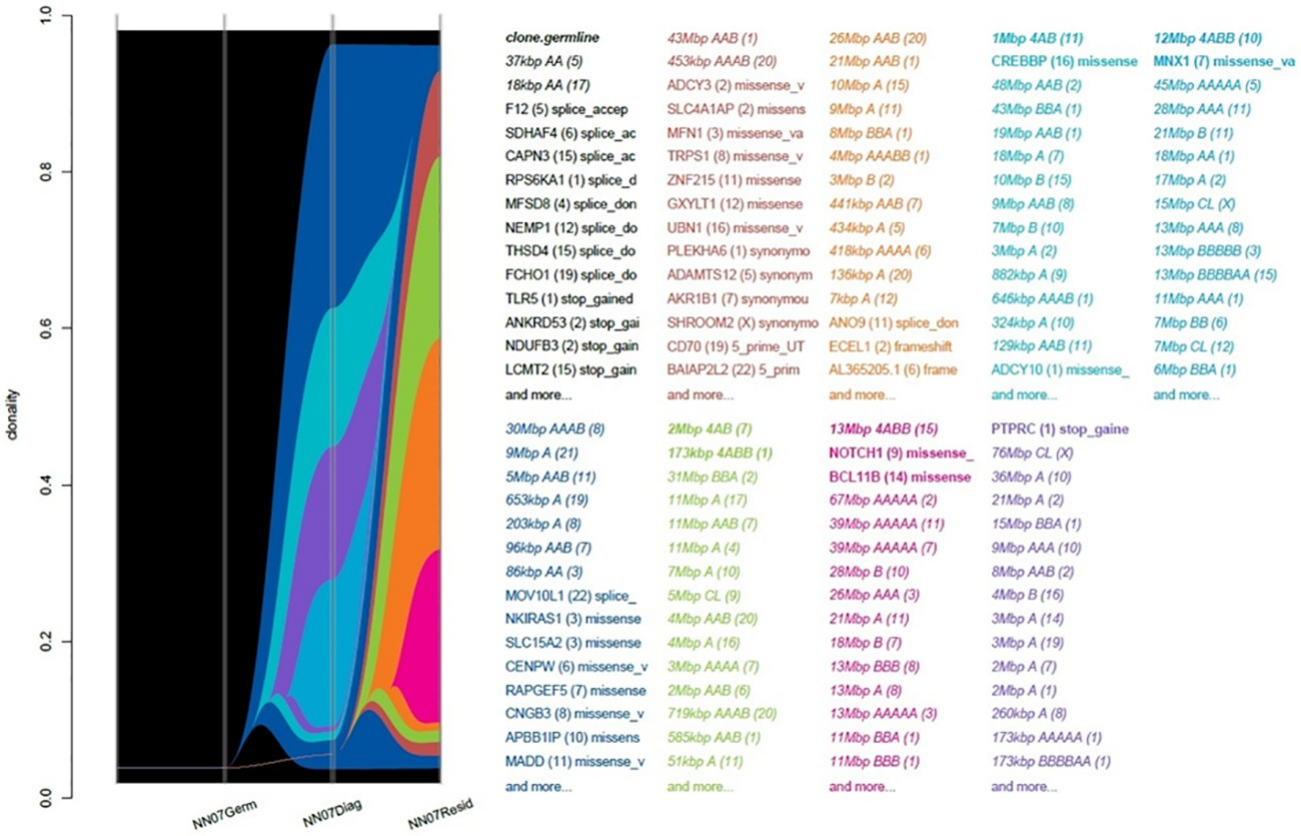
River plot depicting the clonal structure and evolution of diagnostic and residual disease samples from patient N07. The plot demonstrates persistence of one dominant subclone and emergence of four new subclones in the residual disease.

**Fig 6:**
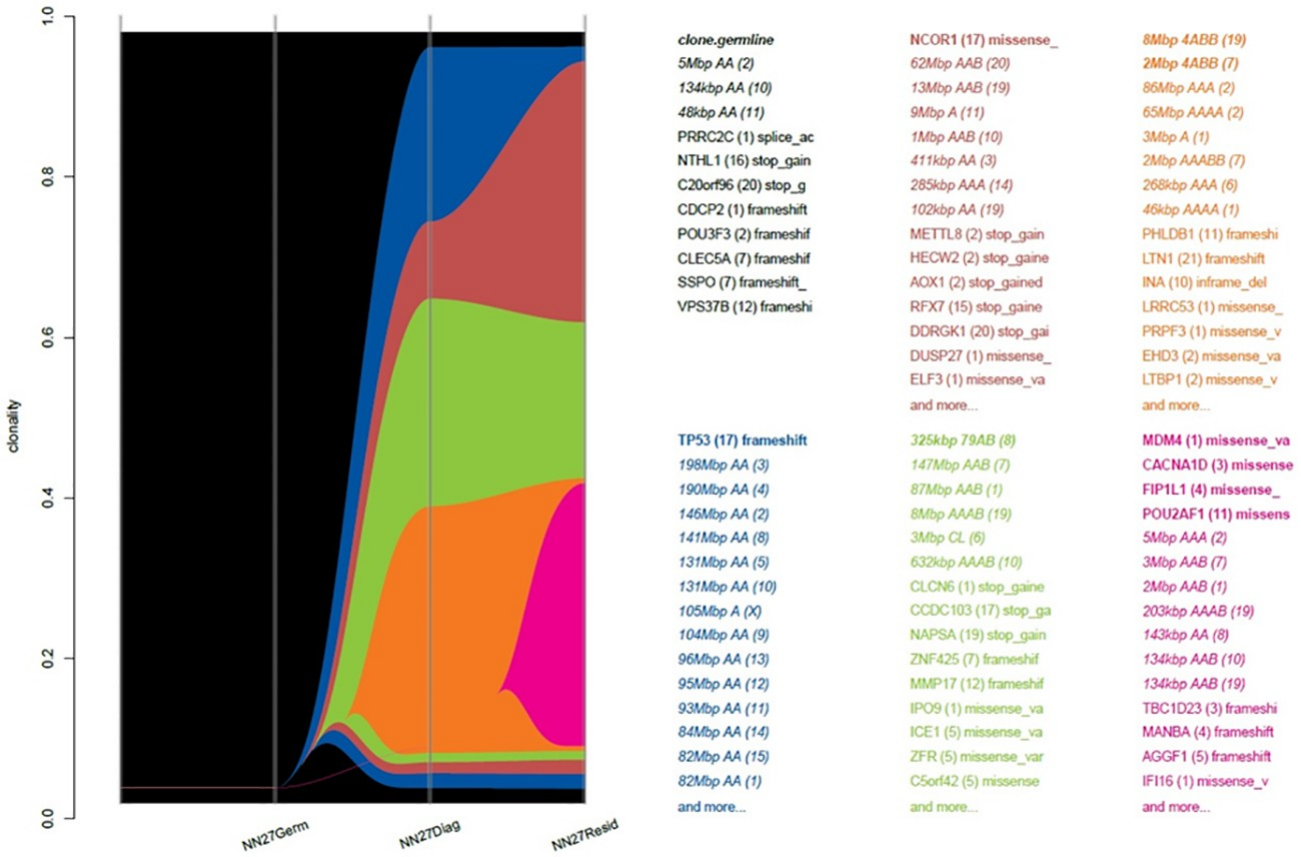
River plot depicting the clonal structure and evolution of diagnostic and residual disease samples from patient N27. The persistence of four subclones (blue, brown, green, orange) and the emergence of a new subclone (pink) despite treatment with neoadjuvant chemotherapy is depicted.

Three patients with residual disease, N07, N08 and N27, developed disease recurrence after surgery. The majority of subclones present in D07 responded to chemotherapy and were undetectable in the residual disease. There was persistence of one dominant clone and emergence of four new subclones, one of which contained alterations in the known breast cancer driver *NOTCH1* (Fig 5). Sample D08 contained only two subclones, one of which responded to chemotherapy and was not detectable in R08. The persistent clone contained alterations in *MED12*, a transcriptional initiator, and *VAV3*, involved in cytoskeletal rearrangements and transcriptional activation, neither of which are implicated in carcinogenesis or actionable targets. Patient NN27 experienced disease progression during neoadjuvant therapy and subsequently died of metastatic breast cancer within months of completing protocol treatment. Consistent with the lack of clinical response and the minimal change in mutation burden or gene pathway alterations, analysis of D27 and R27 samples showed relatively stable populations of subclones present in the diagnostic sample and the emergence of a single new subclonal population in the residual disease (Fig 6). This subclone contained missense mutations in the *TP53* regulator *MDM4*, as well as *FIP1L1*, an oncogenic driver in prostate cancer, *POU2AF1*, a transcriptional activator in Hodgkin’s disease and frameshift mutations in *IFI16*, which modulates *TP53* and RAS/RAF signalling. Other subclones persisting between D27 and R27 were identified by mutations in *TP53* itself and the driver mutation *NCOR1*. Taken together, the mutational burden, gene pathway, mutational signature and clonal evolution analyses illustrate primary resistance to chemotherapy across multiple subclones, making the identification of targets for novel therapies particularly difficult in this case.

## Discussion

As part of this study we have characterised the genomic profiles of a well-defined group of clinically annotated TNBC tumours prior to, and following, neoadjuvant anthracycline/taxane chemotherapy. It has previously been demonstrated that metastatic disease, when compared to its originating primary tumour, is typified by a higher mutation load and the acquisition of new driver mutations, whereas synchronous LN metastases more closely resemble their originating lesion [11, 37]. Underscoring the heterogeneity of TNBC, in our study, the variation observed between paired diagnostic and residual disease samples was markedly less than the variation between tumours from different patients. In our cohort, gene pathways and mutational signatures operating in the diagnostic specimens were mostly present in the matched residual disease, indicating a degree of *de novo* resistance. Our analysis reveals that the residual disease, although altered, still bears close resemblance to pre-treatment tumour. This may simply be a function of the short (approximately 6–7 month) time interval between biopsy and surgical resection, but also suggests further acquisition of genomic alterations are necessary for the development of metastatic disease.

Signature 3 was detected in the majority of samples from our cohort. The presence of this signature signals bi-allelic loss of *BRCA1/2* or other alterations causing HRD [35]. Although we did not observe an association between the presence of Signature 3 and response to anthracycline/taxane chemotherapy, three previous retrospective studies have shown some association between HRD, defined by *BRCA* mutation, *BRCA* methylation or HRD score, and response to standard neoadjuvant anthracycline-based chemotherapy [12, 38, 39]. It has been established in both breast and ovarian cancer that HRD defines a subset of tumours responsive to platinum chemotherapy and poly-ADP ribose polymerase (PARP) inhibition [40–42]. In breast cancer, methods for defining HRD have focused on the detection of germline and somatic mutations or epigenetic silencing of HRD genes and copy number alterations (CNA), large-scale transitions or loss of heterozygosity in the tumour genome [43]. As yet, no one method for identifying HRD has emerged as clearly superior to any other. Mutational signatures that highlight INDEL and CNA alterations have now been developed, and a composite signature, HRDetect, which incorporates base substitution, CNA and INDEL signatures, has been validated as a marker of HRD and sensitivity to platinum chemotherapy [44, 45]. Due to our small number of samples we were not able to perform an analysis using HRDetect. However, with the presence of Signature 3, four of the five patients with residual disease bore a marker of HRD. This raises the question of whether these patients may have benefited from intensification of chemotherapy with the addition of a platinum agent. Mutational signature tools like HRDetect have considerable potential for clinical use and warrant further investigation in clinical trials. If they emerge as a front runner amongst the various methods currently available for identifying HRD they may help target a subset of TNBC patients who could benefit from a DNA damaging agent such as a platinum or PARP inhibitor as part of their therapy.

Our analysis of gene and gene pathway alterations permits several observations regarding *TP53*, ATM signalling and genes encoding subunits of the SWI/SNF complex. Mutations in *TP53* are the most common recurrent alterations found in TNBC, but their relationship to chemotherapy response remains unclear. Although pre-clinical data demonstrates an association between *TP53* mutation and anthracycline resistance, clinical studies have produced conflicting results [11, 46, 47]. Our observation is consistent with more recent studies showing no relationship to response or resistance.

ATM signalling is a crucial biological pathway by which the cancer cell mediates its response to double-stranded DNA breaks and enacts DNA repair [48]. As such, alterations in this pathway are a potential cause of HRD. This pathway was enriched for functional mutations in three of the five patients with residual disease, although sample size limits insight into whether ATM signalling alterations differentiate responders from resistant tumours. These alterations are, however, further evidence of HRD in these tumours and again raise the question of whether there would be a benefit to intensifying treatment with the addition of a platinum drug in these patients.

The SWI/SNF chromatin regulating complex is one of the most common mediators of carcinogenesis, with mutations, translocations and deletions present in approximately 20% of all cancers [49]. Recently, Yates et al. [11] have reported the emergence of SWI/SNF complex mutations in breast cancers resistant to taxane chemotherapy, an observation mirrored in a separate study of ovarian tumours [50]. In our cohort, *ARID1A* and *ARID1B* mutations were identified in the residual disease of two patients, R09 and R07, that were not found in the matched diagnostic samples. Through its effects on chromatin remodelling, gene silencing and cell differentiation, the SWI/SNF complex acts in multiple ways as a tumour suppressor [51]. The *ARID1A* and *ARID1B* subunits have an antagonistic effect on cell cycle progression and mutations with functional impact in these subunits are potential targets for new therapies [51, 52]. Specifically, *ARID1A* deficient cells, due to defective cell cycle regulation, are reliant on *ATR* checkpoint activity. Pre-clinical work has identified *ARID1A* deficiency as a biomarker for the efficacy of *ATR* inhibitors, and in ovarian cancer cells, the multi-kinase inhibitor dasatinib [52, 53]. Alterations in the SWI/SNF complex genes have now also been reported to confer resistance to immunotherapies in melanoma and renal cell cancers [54, 55]. Alterations in *ARID1A* were also found in two chemosensitive samples, D20 and D21. These mutations alone may not discriminate between tumours that will achieve pCR and those that will not, but their presence in resistant disease may identify cancers sensitive to a novel targeted agent. Defects in the SWI/SNF complex in chemoresistant tumours merit further investigation, most importantly as there are now methods of targeting these defects emerging from pre-clinical studies [52, 53].

Prior studies have shown the origin of breast cancer metastases can be traced to subclones present in the primary tumour, but it is not yet possible to identify prospectively which subclone provides this metastatic seed [6, 11, 37]. Comparative examination of the subclonal structure of primary and residual disease allows exploration of likely candidate clones that may give rise to metastatic spread due to their ability to persist or emerge under the selective pressure of neoadjuvant chemotherapy. Identification of these clones and personalising further therapy based on any targetable drivers, mutational processes or pathways they may contain could provide an opportunity to eradicate the disease prior to the development of incurable metastatic spread. Identifying which patients with residual disease are at sufficiently high risk of developing metastatic disease and would benefit from further intensification of therapy requires significant further research.

## Conclusions

Our analyses of primary TNBC pre- and post-neoadjuvant chemotherapy reiterates the known genomic heterogeneity of this disease. Acknowledging the limitations imposed by small sample size, a few hypothesis-generating observations can be made. Though few commonalities are identified, both our gene pathway and mutational signature analyses detected alterations indicative of HRD in both primary and residual disease. Clearly defining HRD in TNBC, potentially with the use of mutational signatures, may enable us to target patients with residual disease that are most likely to benefit from prospective studies investigating the addition of DNA damaging agents such as platinum or PARP inhibitors to standard therapy. This project reinforces our understanding that TNBC, in genomic terms, is a subgroup comprised of often only loosely related cancers and mechanisms of resistance vary from tumour to tumour. In future, with further advances in the cost and efficiency of WES technologies, a precision medicine approach may be used to identify individual patients with targetable tumour defects.

## Supporting information

S1 Table Primer sequences for variant validation. (DOCX)

S2 Table Gene pathways significantly enriched in diagnostic samples. (DOCX)

S1 File Consort checklist. (DOC)

S2 File NEONAB protocol. (PDF)

S3 File NEONAB data variants, unfiltered. (XLSX)

S4 File NEONAB data variants, filtered. (XLSX)
