# Supplementary material for: Tailored NEOadjuvant epirubicin, cyclophosphamide and Nanoparticle Albumin-Bound paclitaxel for breast cancer: The phase II NEONAB trial—Clinical outcomes and molecular determinants of response
Source: PLoS One. 2019 Feb 14;14(2):e0210891. doi: 10.1371/journal.pone.0210891 (PMC6375556; doi:10.1371/journal.pone.0210891)
Supplement: S1 Table — (DOCX) [file pone.0210891.s001.docx]

**S1 Table: Primer sequences for variant validation**

| **PRIMER** | **SEQUENCE** |
| --- | --- |
| AM362_KMT2Dex34-R1 | GCA GGT CAA ACT CGT CTC CA |
| AM361_KMT2Dex34-F1 | CTT CCC CCA CAG AAA CCC TC |
| AM360_ARID4Bex17-R1 | AGC TAA AAC TCA AGT TAC CTC ACA T |
| AM359_ARID4Bex17-F1 | CCT GGA ACA ATC ACA GCC A |
| AM358_PDLIM3ex8-R1 | ATC TGC ACA ATC CTT CTG CCC |
| AM357_PDLIM3ex8-F1 | CTC GTT TTC TTC CCC AAC AGC G |
| AM356_ATRNex10-R1 | GGC AAA AAG ATG CCT GAA GAG A |
| AM355_ATRNex10-F1 | TGG TTG TGC TTG CAT TAG CAG |
| AM354_LRP8ex1-R1 | GCT TTG TTG GTG GCT AGG G |
| AM353_LRP8ex1-F1 | CGA GCG TCA CCG AAC CT |
| AM352_CASP8ex12-R1 | GTG TAA CAG TGA GGA GGG CTG |
| AM351_CASP8ex12-F1 | AGG CTT GTC AGG GGG ATA AC |
| AM350_MSH3ex1-R1 | TCA CCC AAT TTG TAG CCC CG |
| AM349_MSH3ex1-F1 | GCG GTT TTG AGC CGA TTC TT |
| AM346_STAMPBex4-R1 | TTT TGA GTT TCT GGT CTT TGC CC |
| AM345_STAMPBex4-F1 | TGT TGC AGT CAC CAA CTT GAC |
| AM344_TRIOex19-R1 | CAA CGT GCA GGC TCC TTT TG |
| AM343_TRIOex19-F1 | ACC AGC AGG TGG CCA GAT A |
| AM342_FAT4ex9-R1 | TGT CCT TGT CCA TTG CCG AA |
| AM341_FAT4ex9-F1 | CGG ACC ACT AAA CGG AGC TT |
| AM340_ZMAT3-R1 | AGG GCA AGT TGA CAA AAG GC |
| AM339-ZMAT3ex6-F1 | ATC TCT TGC TTT AGC AGG TCC TTA C |
| AM338_GABBR2ex7-R1 | AAC AGC CCC TCT TTT GCT TT |
| AM337_GABBR2ex7-F1 | CTG ATG TCC CTG CCG AGT A |
| AM336_DAPK1ex3-R1 | CTG GAA CGA AGA TGC CAG C |
| AM335_DAPK1ex3-F1 | CAG TGG ACA GTT TGC GGT TG |
| AM334_PTPRQex27-R1 | TCT CCT AAT GCC TTC TCT CTT CTC |
| AM333_PTPRQex27-F1 | TAA GCT GGA GTG AAC CTG CTG |
| AM332_LRRK2-R1 | AAT GAG AGC TGT CCT CTG TCG |
| AM331_LRRK2ex44-F1 | ACA GTT TTA GGT TTT GCT TGA CAG A |
| AM330_TP53-R1 | CAA CCA GCC CTG TCG TCT |
| AM329_TP53ex7-F1 | GAG GTG CTT ACG CAT GTT TGT |
| AM328_ARID1Bex1-R1 | GCT TCG CCA ACG GTT TTC AG |
| AM327_ARID1Bex1-F1 | CCC CGT CAC GAA CTC AAC AT |
| AM326_SETD1Aex8-R1 | GCT CCA AGG AGT TCA CAA AGT CA |
| AM325_SETD1Aex8-F1 | GAT GAG AAA GTA GGG CTT GGG T |
| AM324_SLC22A4ex1-R1 | CGC AGG GAG TCT CAA GGG |
| AM323_SLC22A4ex1-F1 | GGA CTA CGA CGA GGT GAT CG |
| AM322_NCOR1ex31-R1 | GAT AAC TCA CAG GGG TCC TCC |
| AM321_NCOR1ex31-F1 | GCC AGA TGA CTT TGC TAC CTG T |
| AM320_INPP4Aex5-R1 | GCT TCA GCT ACA GAT GCA CAA G |
| AM319_INPP4Aex5-F1 | ACT TCC CTG TGA CAA CTG ACG |
| AM318_INF2ex13-R1 | CAC TCA CTT TCG CAG GCA G |
| AM317_INF2ex13-F1 | AGT CCT TCC CCT CAA AGT GTG |
| AM316_INF2ex15-R1 | CTA GAC GGA GCC CTG AGA CA |
| AM315_INF2ex15-F1 | AGC TGA TCC TGA GAA TTG GGA A |
| AM314_SF1ex9-R1 | AAG AGG TCA ATG CAA GGG AAC A |
| AM313_SF1ex9-F1 | AAG GCA GTG GAA CAG GTG AG |
| AM312_SOX9ex1-R1 | TAC CTC CAG AGC TTG CCC AG |
| AM311_SOX9ex1-F1 | CGA GCC CGA TCT GAA GAA GG |
| AM310_WDPCPex12-R1 | GGA GAT GGA CTG TTA GGA AAC ACA |
| AM309_WDPCPex12-F1 | TGC AAA ACC TGA CTG ACT GC |
| AM308_ARID5Bex10-R1 | GGT AGG TTT GTG CGG GTT CT |
| AM307_ARID5Bex10-F1 | CCT CTA CAG ACA CAC CGA GC |
| AM306_FSHRex10-R1 | AGG GAG GCA GAA ATG GCA AA |
| AM305_FSHRex10-F1 | CCA TCT TTG GCA TCA GCA GC |
| AM304_GATAD1ex5-R1 | TAC AAA TGG TTG GCA ACT GAT TCC |
| AM303_GATAD1ex5-F1 | TAT TTA ACC TTT CCC TTG GCT GC |
| AM302_DCAF13ex10-R1 | GGT GAG ATG AGA GCA GAA GTC A |
| AM301_DCAF13ex10-F1 | AGT GCT TGC AGA GCT AGT GT |
| AM300_PTK2-R1 | TTC TGT CCT TTT CCT CTT CTG GA |
| AM299_PTK2ex7-F1 | GAT GCC TCT CCC CCA GTT TT |
| AM298_RAB3GAP1ex15-R1 | AAT TCC TGC CAG AGG TGT G |
| AM297_RAB3GAP1ex15-F1 | AAA TCT TGA AAA TGT GGG ACT TCC T |
| AM296_TP53ex16-R1 | GGG ACA GCT TCC CTG GTT AG |
| AM295_TP53-F1 | TCT TGT TCC CCA CTG ACA GC |
| AM294_CICex11-R1 | AAT CTC CCC CTC TCT CAA GC |
| AM293_CICex11-F1 | CAT ACT GTT CCC TCC ACT CCC |
| AM292_ZAP70ex11-R1 | CACAGGGGCAGAGGCTTAC |
| AM291_ZAP70ex11-F1 | CCTGGGAACTTGGCTAGTCTT |
| AM290_BRCA2ex11-R1 | AAAAGCCCCTAAACCCCACTT |
| AM289_BRCA2ex11-F1 | AGTGCCTGAAAACCAGATGACT |
| AM288_FBN2ex23-R1 | GGTTTTGGACTAGCCACTGCTT |
| AM287_FBN2ex23-F1 | GAAGTAGGACCACACCTGCT |
| AM286_FAT4ex1-R1 | CCCACTCACATAGCTTCCCG |
| AM285_FAT4ex1-F1 | GTG CCG AAC CTG AGC CTA AT |
| AM284_MTMR14ex17-R1 | CCCCATCTTGAGACCCTAGC |
| AM283_MTMR14ex17-F1 | CACAGAGTGTCCTCTGGAACC |
