# Supplementary material for: Tailored NEOadjuvant epirubicin, cyclophosphamide and Nanoparticle Albumin-Bound paclitaxel for breast cancer: The phase II NEONAB trial—Clinical outcomes and molecular determinants of response
Source: PLoS One. 2019 Feb 14;14(2):e0210891. doi: 10.1371/journal.pone.0210891 (PMC6375556; doi:10.1371/journal.pone.0210891)
Supplement: S2 Table — (DOCX) [file pone.0210891.s002.docx]

**S2 Table: Gene pathways significantly enriched in diagnostic samples**

| Sample | Category | Pathway Term | Gene Count | P-Value |
| --- | --- | --- | --- | --- |
| D07 | KEGG_PATHWAY | Transcriptional misregulation in cancer | 5 | 0.0056 |
| D08 | BIOCARTA | ATM signalling pathway | 2 | 0.026 |
| D27 | BIOCARTA | ATM signalling pathway | 3 | 0.024 |
| D27 | BIOCARTA | Cell cycle | 3 | 0.033 |
| D27 | BIOCARTA | Control of gene expression by vitamin D receptor | 3 | 0.036 |
| D27 | BIOCARTA | Cell cycle | 3 | 0.046 |
| D27 | KEGG_PATHWAY | Chronic myeloid leukaemia | 6 | 0.00054 |
| D27 | KEGG_PATHWAY | Hepatitis B | 7 | 0.0023 |
| D27 | KEGG_PATHWAY | Prostate cancer | 5 | 0.0092 |
| D27 | KEGG_PATHWAY | Pathways in cancer | 10 | 0.011 |
| D27 | KEGG_PATHWAY | Thyroid hormone signalling pathway | 5 | 0.022 |
| D27 | KEGG_PATHWAY | Glioma | 4 | 0.023 |
| D27 | KEGG_PATHWAY | Pancreatic cancer | 4 | 0.023 |
| D27 | KEGG_PATHWAY | p53 signalling pathway | 4 | 0.025 |
| D27 | KEGG_PATHWAY | Cell cycle | 5 | 0.029 |
| D27 | KEGG_PATHWAY | HTLV-I infection | 7 | 0.033 |
| D27 | KEGG_PATHWAY | Proteoglycans in cancer | 6 | 0.039 |
| D29 | BIOCARTA | Tumour suppressor Arf inhibits ribosomal biogenesis | 3 | 0.03 |
| D29 | BIOCARTA | ATM signalling pathway | 3 | 0.04 |
| D29 | KEGG_PATHWAY | mTOR signalling pathway | 4 | 0.014 |
| D29 | KEGG_PATHWAY | PI3K-Akt signalling pathway | 8 | 0.029 |
