# Supplementary material for: Tailored NEOadjuvant epirubicin, cyclophosphamide and Nanoparticle Albumin-Bound paclitaxel for breast cancer: The phase II NEONAB trial—Clinical outcomes and molecular determinants of response
Source: PLoS One. 2019 Feb 14;14(2):e0210891. doi: 10.1371/journal.pone.0210891 (PMC6375556; doi:10.1371/journal.pone.0210891)
Supplement: S2 File — (PDF) [file pone.0210891.s004.pdf]

ALCC 12.01

Tailored Neoadjuvant Epirubicin And Cyclophosphamide (EC) And Nanoparticle Albumin Bound (NAB) Paclitaxel For Newly Diagnosed Breast Cancer

NEONAB

Version Number: Version 5.0
Date: 24 June 2014

Principal Investigator: Dr Mustafa Khasraw

Co-Investigators:
Prof. David Ashley
Dr Madhu Singh
Dr Adam Broad
Dr Karen White
Dr Inger Olesen
Prof Wei Duan
Dr Sarah Shigdar
Mr Conrad Brandt
Dr Linda West
Dr Kimberly Price
Mr Gregory Mitchell
Dr Graham Pitson
Dr Michael Francis

**PROTOCOL APPROVAL**<br>Protocol Author: Dr Mustafa Khasraw<br><br>Signed:Date:

Haematology and Oncology Trials Team
Andrew Love Cancer Centre
The Geelong Hospital, Barwon Health
70 Swanston Street
Geelong, VIC 3220
AUSTRALIA
E-mail: m.khasraw@deakin.edu.au

Telephone: +61-3-4215 2764
Facsimile:+61-3-4215 2835

Study Statistician:
Sujata Patil, Biostatistician
Department of Epidemiology and Biostatistics
Memorial Sloan Kettering Cancer Center
307 E.63rd Street, 3rd floor,
New York, NY 10065 USA
Tel: + 1-646 735 8182
E-mail: patils@mskcc.org

Table of Contents:

1 Abbreviations 5
2 Synopsis 6
3 Background and Scientific Rationale for Study: 7
3.1 Breast Cancer 7
3.2 Primary SystemicTherapy (PST) 7
3.3 Nanoparticle albumin-bound paclitaxel 7
3.4 Epirubicin and Cyclophosphamide 8
3.5 (Neo)Adjuvant Treatment of Breast Cancer 8
3.6 Ki-67 labelling Index and Oncotype DX Recurrence Score (RS) 8
4 Translational Research 9
4.1 Breast Cancer Stem Cells 9
4.2 Aptamers 9
5 Intervention 9
5.1 Chemotherapy Regimens: 9
5.1.1 Exploratory cohort 10
5.2 Surgery 10
5.3 Radiotherapy and hormone therapy 10
6 Dose modification and delay 10
6.1 Dose Modification and Delay guidelines: 10
7 Eligibility 13
7.1.1 Study Description: 14
7.1.2 Design 14
7.1.3 Statistical Aspects: 14
7.2 Translational Endpoints: 15
*7.2.1 NQO1*2 genotype (P187S) status 15*
7.2.2 Pre- and Post Chemotherapy tissue Characteristics 15
7.2.3 Optional translational research: 15
8 Details of the Translational Experiments 15
8.1 Establishing primary breast cancer stem cell xenograft models. 15
8.2 Development of a novel cancer stem cell-targeting nanoparticle 17
9 Clinical endpoints 18
9.1 Evaluation of Clinical Activity 18
9.2 Endpoints and assessments: 18
9.2.1 Histopathology assessment 18
9.2.2 Assessment of clinical tumor response 18
9.2.3 Central imaging review 18
9.2.4 Surgery 19
9.3 Primary Endpoint: 19
9.3.1 Histopathological sampling and review 19
9.4 Secondary end points: 19
9.5 Translational endpoint: .... **Error! Bookmark not defined.**
10 Schedule of assessments 20
10.1 Pre-treatment 20
10.2 Evaluations during treatment 20
10.3 Evaluations after treatment 20
10.3.1 Patient related outcome measures 23
11 Patient safety (Monitoring of Serious Adverse Events) 23
11.1 Adverse events 23
11.2 Reporting of Serious Adverse Events (SAE) 23
11.3 Other events to be treated as SAEs 24
11.3.1 Exposure to drug during pregnancy/lactation 24
11.4 Events not treated as SAEs 24
11.5 Methods of recording and assessing toxicity (Adverse Events) 25
11.6 Recording and assessing SAEs 25
11.7 Causality 26
11.8 Duration 26
11.9 Action Taken 26
11.10 Outcome 26

11.11 Abnormal Laboratory Values 26
11.12 Females of Childbearing Potential: 26
11.13 Expedited Reporting of Adverse Events 27
11.13.1 Reporting to Regulatory Authorities and the Ethics Committee 27
11.14 Immediate reporting by Investigator to the Haematology and Oncology Trials Team and the Haematology and Oncology Trials Team to Celgene 27
11.15 Safety instructions specific to the study 28
12 Ethical principles 28
12.1 Informed consent 28
12.2 Confidentiality 28
12.3 Study Monitoring 28
12.4 Responsibilities of the investigators 28
12.5 Data collection and quality assurance 28
12.5.1 Data Collection Forms and database 28
12.5.2 Monitoring 29
12.5.3 Protocol Deviations and violations 29
12.6 The use and completion of case report forms (CRFs) 29
12.7 Record retention in investigator centres 29
12.8 Publications 29
12.9 Protocol amendments 29
13 References: 31

1Abbreviations

ADR	Adverse drug reaction
AE	Adverse event
BMI	Body mass index
BP	Blood pressure
CNS	Central nervous system
COX2	Cyclooxygenase-2
CRF	Case report form
CT	Computerised tomography
CTCAE (v4.0)	NCI common terminology criteria for adverse events version 4.0
CVD	Cardiovascular disease
DNA	Deoxyribonucleic acid
DSMB	Data Safety Monitoring Board
EC	Epirubicin and Cyclophosphamide
ECG	Electrocardiogram
ER	Estrogen Receptor
EUC	Electrolytes, Urea, Creatinine
FACS	Fluorescence-activated cell sorting
FBE	Full blood examination
FISH	Fluorescence In Situ Hybridization
GFR	Glomerular filtration rate
G-CSF	Granulocyte colony-stimulating factor
HER2	Human Epidermal Growth factor Receptor 2
IHC	Immunohistochemistry
ISH	In Situ Hybridization
IV	intravenously
LFTs	Liver function tests
NAB Paclitaxel	Nanoparticle Albumin Bound Paclitaxel
NAD+	Nicotinamide adenine dinucleotide
NCI CTCAE	National Cancer Institute common terminology criteria for adverse events
nCR	Near complete response (only scattered tumor cells left)
NOD mice	Non-obese diabetic (NOD) mice
NSAIDS	Nonsteroidal anti-inflammatory drugs
NQO1	NAD(P)H: quinone oxidoreductase 1
OS	Overall survival
PCI	Prophylactic Cranial irradiation
pCR	Pathologic Complete Response
PET scan	Positron Emission Tomography
PFS	Progression-free survival
PI	Principal Investigator
PgR	Progesterone Receptor
PS	Performance status
PST	Primary Systemic Therapy
PTEN	Phosphatase and tensin homolog
QoL	Quality of Life
RECIST	Response Evaluation Criteria In Solid Tumours
qPCR	Quantitative Polymerase Chain Reaction
RR	Response Rate
RS	Oncotype DX Recurrence Score
SAE	Serious adverse event
SCID mice	Severe combined immunodeficiency mice
SLNB	Sentinel Lymph Node Biopsy
U/S	Ultrasound

2Synopsis

Title

Tailored Neoadjuvant Epirubicin and Cyclophosphamide (EC) And Nanoparticle Albumin Bound (NAB) Paclitaxel for Newly Diagnosed Breast Cancer

Aims

To evaluate tailored PST using with sequential NAB Paclitaxel and EC in early breast cancer.

Objectives

- Overall objective response rate.

Hypothesis

Tailored neoadjuvant chemotherapy with sequential NAB paclitaxel and EC is feasible and achieves high response rates.

Treatment Description

Tailored PST incorporating sequential EC and NAB paclitaxel

Trial Design

Open label phase II clinical trial

Number of Patients

40 patients (15 HER2 positive, 15 Triple negative and 10 HR positive with Oncotype RS > 25) will be evaluable for response. Up to 50 HR positive patients will be screened and patients with Oncotype recurrence score </= 25 will be included as an exploratory cohort but will not be evaluable for response (see study scheme).

Target Population

Women with early breast cancer suitable for primary systemic therapy

Primary Endpoints:

To evaluate overall objective response rate by measuring the rate of pCR in the breast.

Secondary end points:
Secondary Endpoints

- Response rate in breast and axillary lymph nodes.
- Rate of pCR and nCR in the breast
- Rate of breast conservation
- Safety and tolerability
- PFS using Kaplan-Meier methods

Statistical Aspects

The primary endpoint for this study is objective rate of pCR (*proportion of patients achieving pCR*) in the breast at surgery. In previous neoadjuvant studies, RR have ranged from 12 to 30%. Accordingly, we will set the RR rate for the null hypothesis (uninteresting rate) at 30% and for the alternative (worthy of further study) at 50%. For a single stage design, we will require 40 evaluable patients to discern between response rates of 30% and 50%. The type I error is 6% and the power is 87%. If at the end of the study, 17 or more patients have a pCR, the regimen will be deemed worthy of further study.
The response rate with 95% confidence interval will be calculated at the end of the study. Patients who received at least 1 cycle of chemotherapy will be evaluable. PFS will be evaluated using Kaplan-Meier methods. Response rates will be calculated with 95% confidence intervals.

3Background and Scientific Rationale for Study:

3.1Breast Cancer

Breast Cancer is a heterogeneous disease. Prognosis and survival rate of breast cancer varies greatly depending on extent of the disease, performance status of the patients and the type of the tumour including the status of oestrogen receptor (ER), progesterone receptor (PR), and Human Epidermal growth factor Receptor 2 (HER2). Expression of the ER and PR confers a better prognosis than the overexpression of HER2 and lack of expression of ER or PR or HER2 overexpression (triple-negative breast cancer (TNBC)) tend to be indicative of a more aggressive cancers with a high growth rate[1]. TNBC occur most frequently in young women and they are highly responsive to conventional chemotherapy but relapse earlier and more frequently than hormone receptor-positive breast cancer.

3.2Primary SystemicTherapy (PST)

Preoperative or neoadjuvant therapy which is also known as primary systemic therapy (PST) followed by surgery and adjuvant radiation therapy (RT) is recommended for patients with locally advanced breast cancer[2]. Studies using PST have demonstrated useful rates of clinical response (cCR) and pathological Complete Response (pCR) rates in the breast alone (ypT_0) and pCR rates in the axillary lymph nodes(ypN0)[3]. Definitions of response vary considerably, however response rates to cytotoxic chemotherapy have been uniformly higher in hormone receptor negative (ER negative) tumors. There is additional improvement in the pCR rate of about 10% with the addition of a taxane[4]. Most large randomized trials comparing PST against identical Adjuvant Systemic Therapy (AST) indicate that these strategies offer equivalent disease free and overall survival outcomes. pCR defined as no invasive and no in situ residuals in breast and nodes can best discriminate between patients with favorable and unfavorable outcomes.[5] For operable breast cancer, PST can be considered as an alternative to AST for patients who are deemed to require mastectomy but who desire less extensive surgery (breast conservation surgery). In patients with large tumours who can technically have a lumpectomy, PST may permit less extensive surgery and result in a better cosmetic result. PST may also be advisable in patients who have medical contraindications to surgery or where delayed surgery is required.

3.3Nanoparticle albumin-bound paclitaxel

Nanoparticle albumin-bound paclitaxel is suggested to achieve a higher intracellular tumour paclitaxel concentration via the albumin-mediated trans-endothelial transport system[6]. Better tolerability and efficacy have been demonstrated when compared to paclitaxel or docetaxel in treatment of metastatic breast cancer[7, 8] NAB-paclitaxel is being evaluated in the adjuvant treatment of patients with breast cancer[9, 10] and in the neoadjuvant setting[11].
The pre-operative setting provides an opportunity to study the early molecular changes that may occur in response to treatment. Alteration of biomarkers between pre- and post-chemotherapy including hormone receptors, the Human Epidermal growth factor Receptor (HER-2) and Ki-67[12, 13] as well as gene pathways[14] are areas of possible exploration in neoadjuvant studies whereby tissue is available for analysis before and after the chemotherapy treatment. Particular patterns of reduction in tumour size on MRI can be predictive of successful response, detecting residual tumour not apparent on mammogram or U/S and in accurate evaluation of tumour volume[15]. Functional imaging biomarkers of response also have potential utility in assessing treatment response[16]. A recent study reported that 4 cycles of adjuvant therapy with the combination of NAB-Paclitaxel and cyclophosphamide, with or without trastuzumab, is feasible and well tolerated in patients with early stage breast cancer. Another small study demonstrated feasibility of NAB-Paclitaxel followed by FEC[17].

3.4Epirubicin and Cyclophosphamide

An anthracycline-containing regimen followed by conventional paclitaxel is amongst the most commonly prescribed adjuvant chemotherapy regimens for early breast cancer. Cyclophosphamide is given in combination with doxorubicin or its epimer epirubicin. Epirubicin achieves similar efficacy results to doxorubicin but causes less cardiotoxicity[18-20].

3.5(Neo)Adjuvant Treatment of Breast Cancer

Although standards of care are varied, adjuvant chemotherapy in 2012 is generally recommended in women with TNBC and HER amplified tumors. In women with hormone receptor positive tumours without HER2 amplification, chemotherapy is less effective and it is reserved for tumours that are large or with extensive nodal involvement and/ or high risk biology. The latter includes young age, presence of lymphovascular space invasion, a high proliferative index (Ki67 expression), lower ER/PR expression, higher Oncotype Dx score and luminal B tumors[21]. Thus there are evolving trends to tailor therapy based on the tumor characteristics indicating perceived risk, patient factors, particularly co-morbid illness and patient preferences as well as prediction of response.

3.6Ki-67 labelling Index and Oncotype DX Recurrence Score (RS)

In breast cancer, immunohistochemical assessment of the proportion of cells staining for the nuclear antigen Ki67 has become a widely used method for comparing proliferation between tumour samples[22]. Potential uses include: prognosis, prediction of relative responsiveness or resistance to chemotherapy or endocrine therapy, estimation of residual risk in patients on standard therapy and as a dynamic biomarker of treatment efficacy in samples taken before, during, and after neoadjuvant therapy[22, 23]. Ki-67 labelling Index has been incorporated as one of the means of identifying tumours subtypes by the 2011 St Gallen International Expert Consensus on the Primary Therapy of Early Breast Cancer[21]. Analysis of gene expression arrays has resulted in the recognition of several fundamentally different subtypes of breast cancer[24]. Using gene expression profile distinction can be made between luminal A and luminal B tumours. While both subtypes could be ER positive, luminal A tumours are unlikely to benefit from cytotoxic chemotherapy. Because it is not always feasible to obtain gene expression array information, a simplified classification, closely following that proposed by Cheang *et al.,* whereby a cut-point for Ki-67 labelling index of <15% was established by comparison with PAM50 intrinsic subtyping to differentiate between luminal A and luminal B tumours[25]. Local quality control of Ki-67 staining is important[21]. There is heterogeneity in the Ki67 index depending on which part of the tumour is biopsied and thus sampling misrepresentation may occur. Therefore, in this study Ki67 will only be used if it is consistent with ER expression i.e., low Ki67 should be associated with strong ER expression and high Ki67 with rather weak ER expression.

The Oncotype Recurrence Score is a validated a 21-gene assay that is now offered as a commercial reference laboratory test (Oncotype DX, Genomic Health Inc. Redwood City, CA). The 21-gene panel includes genes involved in tumour cell proliferation and hormonal response, characteristics that have been reported to be associated with chemotherapy response in general. Oncotype DX not only quantifies the likelihood of breast cancer recurrence in women with node-negative, oestrogen receptor–positive breast cancer, but also predicts the magnitude of chemotherapy benefit [21, 26]. Likelihood of chemotherapy benefit is reported as low (Recurrence Score <18, intermediate RS 18-30 and high >30).[26]
The main utility of Oncotype DX is in the adjuvant setting where it could be a powerful tool to guide decision regarding the role of cytotoxic chemotherapy in hormone positive tumours. In this neoadjuvant study we will employ Oncotype DX to guide decision about use of chemotherapy in patients with tumours that have high Ki67 (>15) but low ER expression and also in the other groups that may have low Ki67 (<15) but high ER expression by IHC.

The goal is to use the best combination, sequence and duration of therapy together with predicting and monitoring response with high fidelity in the individual patient. Further studies are needed to optimize treatment regimens so as to increase pathologic response rates and ultimately survival, with a further goal of reducing risk and adverse events.
This study uses a tailored approach to select treatment involving the choice of NAB Paclitaxel and EC based on the individual patient and tumour characteristics (Figure 1).

4Translational Research

4.1Breast Cancer Stem Cells

It is now widely accepted that our inability to cure cancer is largely due to the presence of a subset of cells within a cancer that constitutes a reservoir of self-sustaining [27]. Current radiation and cytotoxic chemotherapies more effectively destroy the proliferating cells that form the bulk of the tumour, but are largely ineffective against the cancer stem cells (CSC)[28, 29]. Breast cancer was the first solid malignancy from which CSCs were identified[30], via specific cell surface marker proteins CD44 and epithelial cell adhesion molecules (EpCAM)[31, 32]. EpCAM and CD44v6 are among best available, clinically relevant breast cancer stem cell markers for the proof-of-principle work in this project [32] .

4.2Aptamers

Aptamers are short, single-stranded RNA or DNA that fold into specific 3-D structures and bind to their target molecules with high affinity and specificity[33]. Unlike antibodies, aptamers remain structurally stable across a wide range of temperature and storage conditions. They are generally non-immunogenic, nontoxic and are 20 to 25 times smaller than monoclonal antibodies. Thus aptamers offer several advantages for tissue penetration and have shorter circulation time and faster body clearance resulting in a low background noise during imaging and lower radiation dose. In addition, aptamers can be produced rapidly, relatively inexpensively, and with high homogeneity[34, 35].

5Intervention

The study will evaluate the feasibility and safety of tailored primary systemic therapy in the study population.

5.1Chemotherapy Regimens:

Sequential epirubicin/ cyclophosphamide followed by NAB-Paclitaxel. Epirubicin 90mg/m2 and cyclophosphamide 600mg/m2 (EC) IV every 3 weeks for 4 cycles followed by NAB-Paclitaxel weekly (125 mg/m2 IV days 1, 8, and 15) 3 weeks of treatment with one week off (28 day cycle) every 4 weeks for 12 weeks.

The above regimen will be offered to all women with (1) tumours overexpressing HER2 receptor by *in situ* hybridization (ISH), (2) triple negative breast cancer (TNBC) and (3) HER2-negative, ER positive and/ or PgR positive breast cancer and an Oncotype DX recurrence score of > 25.

Women with following tumors overexpressing HER2 will be treated with NAB-Paclitaxel in combination with trastuzumab. Trastuzumab will be administered IV with an initial dose of 8 mg per kilogram of body weight. The second and all subsequent maintenance doses will be 6 mg per kilogram given every 21 days. HER2-positive patients will receive subsequent adjuvant trastuzumab for a total duration of 12 months of neo + adjuvant treatment (i.e. from commencement of primary therapy up to 12 months). Further adjuvant treatment can be given upon investigators discretion.

Chemotherapy dose modifications will be specified based on nadir blood counts and interval toxicity. Chemotherapy could be stopped in cases of unacceptable toxicity, failure to recover haematological or severe non-haematological toxicities after a 2 weeks delay, or progressive disease.

5.1.1 Exploratory cohort

As a parallel exploratory cohort, women with ER positive and PgR positive breast cancer and oncotype DX recurrence score of =/< 25 and HER2-negative status may be treated with ***either*** sequential epirubicin/ cyclophosphamide followed by NAB-paclitaxel ***or*** with hormonal treatment or other appropriate treatments at the discretion of the treating physician. This cohort may enrol onto other appropriate clinical trials.

The following data should be collected for patients on the exploratory cohort:

- Treatment details including dosage details i.e. start and stop dates
- Baseline data ***the following items are optional:*** MRI, haematology, biochemistry, liver function tests, vitamin D3 level, 12-lead ECG, ECHO/MUGA test
- If patient is recommended for surgery after treatment, the pathological response should be captured

5.2 Surgery

Mastectomy, lumpectomy, or quadrantectomy with axillary lymph node procedure (sentinel node or dissection) will be performed 3 to 6 weeks after the last chemotherapy administration. In case of complete clinical and radiologic response after neoadjuvant chemotherapy, excision of the tumor bed and axillary lymph node procedure will still be performed.

5.3 Radiotherapy and hormone therapy

Adjuvant radiotherapy will be administered according to the investigator's discretion. Hormone-receptor-positive patients will receive adjuvant endocrine therapy (either tamoxifen 20 mg daily or an aromatase inhibitor depending on menopausal status) for 5 years after chemotherapy completion.

6 Dose modification and delay

If possible, toxicities should be managed symptomatically.

6.1 Dose Modification and Delay guidelines:

Chemotherapy (EC or NAB paclitaxel) will be withheld for any toxicity of grade >/= 2 on the planned day of therapy except for an absolute neutrophil count (ANC) of >/= 0.9 x 10^9/L if neutropaenia has been documented granulocyte colony stimulating factor (G-CSF) should be added. .

If a patient developed grade 3 febrile neutropenia or a grade 3 infection with or without neutropenia, the dose of albumin-bound paclitaxel will be maintained and supported with G-CSF on days 2-7 of each subsequent weekly dose.

Grade 4 infections or febrile neutropenia require a dose reduction and subsequent administration of G-CSF.

Grade ≥ 3 nonhematologic toxicity require a 20% dose reduction.

NAB paclitaxel is to be administered with grade 1 neuropathy but to be withheld for grade ≥ 2

neuropathy on the day of planned therapy.

Patients with transient grade 3 neuropathy could continue therapy when it had improved to grade ≤ 1 but are required to have a dose reduction.

EC is not held or adjusted for persisting neuropathy from albumin-bound paclitaxel (Her2 positive).

Trastuzumab is to be continued if chemotherapy was delayed.

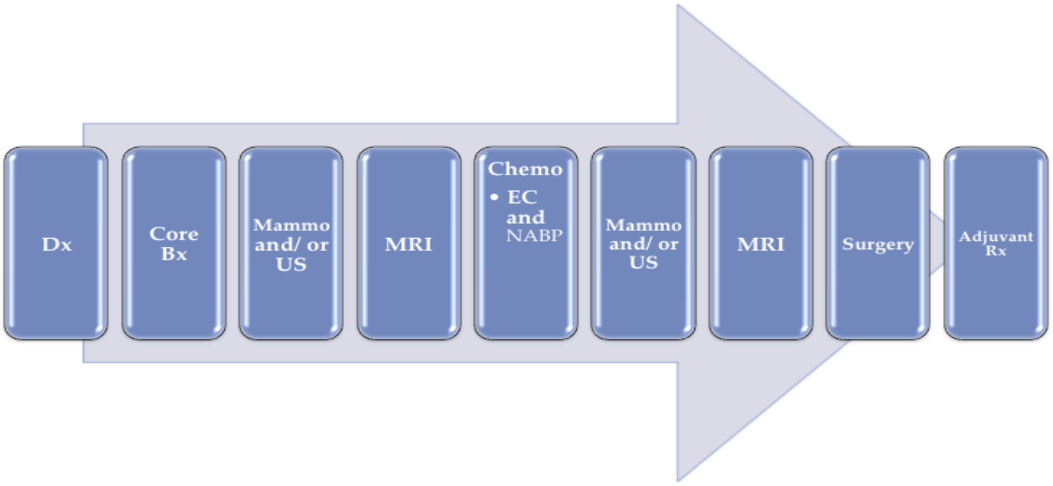

Figure 1; Clinical steps in the study Dx: Diagnosis; Bx: Biopsy; US: Ultrasound; Rx: Treatment; EC: Epirubicin and Cyclophosphamide; NABP: Nanoparticle Albumin Bound Paclitaxel. ***Note that the exploraory cohort is not required to have MRI imaging performed, only standard of care imaging i.e mammogram and/ or ultrasound.***

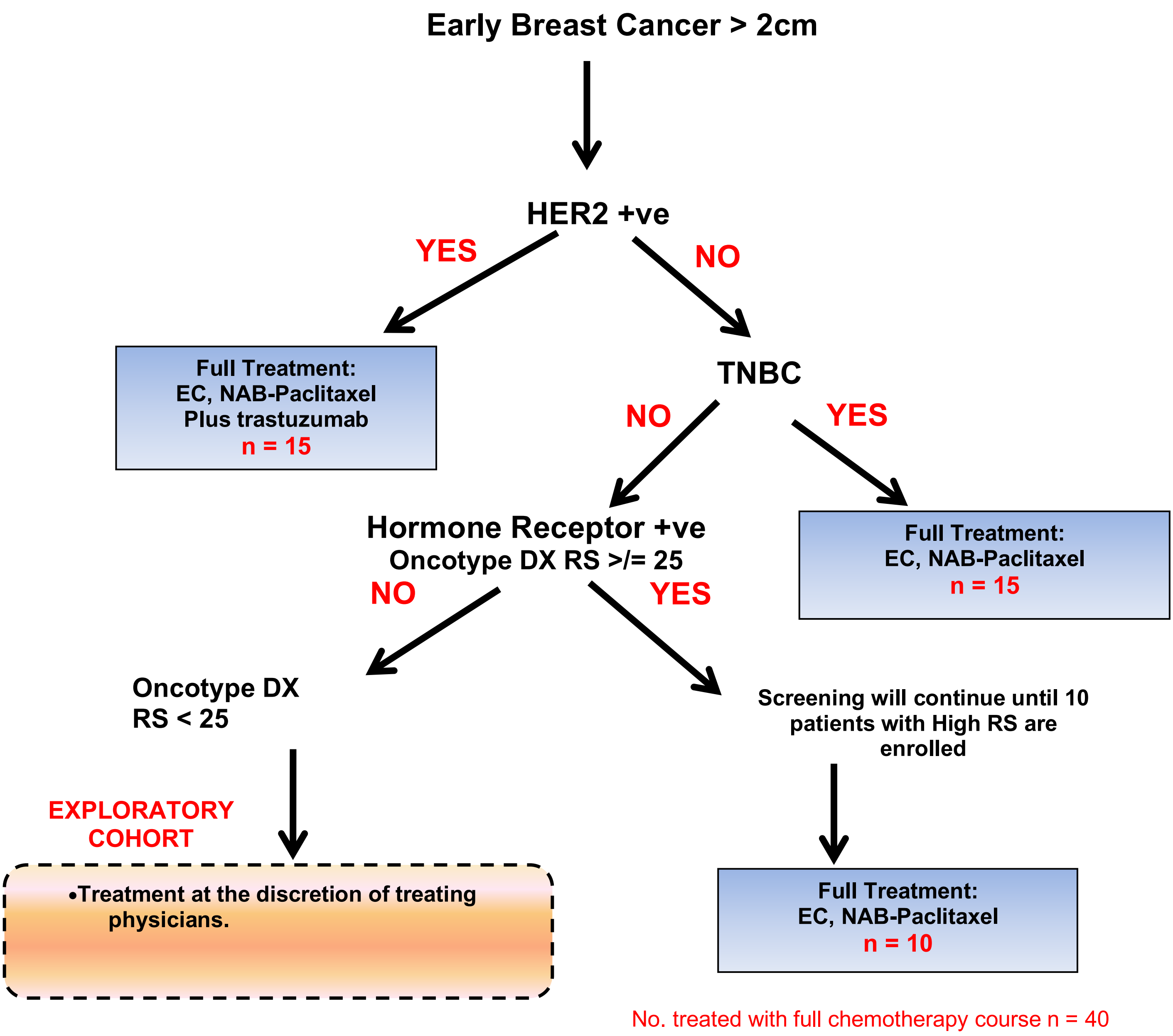

Figure 2.Scheme of treatment options by tumour type. Note "full treatment" patients are the intent-to-treat evaluable population (n =40). Treatment is as follows: **EC**- Epirubicin 90mg/m2 and Cyclophosphamide 600mg/m2 (IV) Q3 wk x 4 followed by **NABP**- Nanoparticle Albumin Bound Paclitaxel. (125mg/m2 IV days 1, 8, 15 Q4 wk) for 12 weeks. Hormonal treatment will be tamoxifen or aromatase inhibitor. We will employ Oncotype DX to guide decision about use of chemotherapy in patients with ER or PR positive tumours. Likelyhood of chemotherapy benefit is reported as low (Recurrence Score <25, high >25).[26] Up to 50 ER+ HER2- patients will receive the Oncotype DX assay to stratify patients, with the anticipation that 10 patients will have Recurrence Score of 25 or greater and be enrolled into the trial. Those with Recurrence Score results under 25 will be treated at the physician's discretion. The Recurrence Score is used only as a patient stratifier in ER+, HER2- patients, there will be no evaluation of the performance of the Oncotype DX assay in this small study.

7Eligibility
Inclusion Criteria:

- The patient must have consented to participate and must have signed and dated an appropriate approved consent form.
- Female 18 Years and older
- The ECOG performance status must be 0 or 1
- The diagnosis of invasive adenocarcinoma of the breast must have been made by core needle biopsy or limited incisional biopsy.
- Patients must have tumor diameter >2 cm measurable at least clinically; by physical exam, unless the patient has inflammatory breast cancer, in which case measurable disease by physical exam is not required or ultrasonographic staging (T2, T3 or T4 a, b, c tumours with any clinical node status N0–N2).
- LVEF assessment by 2-D echocardiogram or MUGA scan performed within 3 months prior to study entry must be greater or equal to 50%.
- Adequate haematological, renal and hepatic function (neutrophils >=2 × 10^9/L, platelets ≥100 × 10^9/L, hemoglobin >=100g/L, total bilirubin ≤ 1.5 x upper limit of normal (ULN), aspartate aminotransferase and alanine aminotransferase ≤1.5 × ULN, alkaline phosphatases ≤2.5 ULN, creatinine ≤ 1.5 ULN).
- Negative pregnancy test in women of a child-bearing age.

Exclusion Criteria:

- Severe cardiovascular, hepatic, neurologic or renal comorbid conditions
- Primary surgical treatment of the tumor or excisional biopsy or lumpectomy performed prior to study entry.
- Surgical axillary staging procedure prior to study entry.
- Definitive clinical or radiologic evidence of metastatic disease.
- History of ipsilateral invasive breast cancer regardless of treatment or ipsilateral DCIS treated with RT.
- Non-breast malignancies unless the patient is considered to be disease-free for 5 or more years prior to study entry and is deemed by her physician to be at low risk for recurrence. Patients with the following cancers are eligible if diagnosed and treated within the past 5 years: carcinoma *in situ* of the cervix, melanoma *in situ*, and basal cell and squamous cell carcinoma of the skin.
- Previous therapy with anthracyclines or taxanes for any malignancy.
- Treatment including RT, chemotherapy, and/or targeted therapy, administered for the currently diagnosed breast cancer prior to study entry.
- Continued therapy with any hormonal agent such as raloxifene, tamoxifen, or other SERM.
- Any sex hormonal therapy, e.g., birth control pills and ovarian hormone replacement therapy
- History of hepatitis B or C.
- Sensory/motor neuropathy greater or equal to grade 2, as defined by the current version of the NCI's CTCAE.
- Pregnancy or continuing lactation at the time of study entry.
- Use of any investigational agent within 4 weeks prior to enrollment in the study.

7.1.1Study Description:

7.1.2Design

Open label multicentre phase II study of neoadjuvant EC followed by NAB paclitaxel in patients with early breast cancer.

Hypothesis:

Tailored neoadjuvant chemotherapy with sequential NAB paclitaxel and EC is feasible and achieves high response rates.

Population: Women with newly diagnosed breast cancer who are eligible for PST.

Study Sites: Barwon Health and affiliate hospitals (SJOG Geelong, Warrnambool) and other interesting sites.

7.1.3Statistical Aspects:

The primary endpoint for this study is response in the breast at surgery *(proportion of patients achieving pCR)*. In previous neoadjuvant studies, pCR have ranged from 12 to 30%.

Accordingly, we will set the RR rate for the null hypothesis (uninteresting rate) at 30% and for the alternative (worthy of further study) at 50%. For a single stage design, we will require 40 evaluable patients to discern between pCR rates of 30% and 50%. The type I error is 6% and the power is 87%. If at the end of the study, 17 or more patients have pCR, the regimen will be deemed worthy of further study. We expect to recruit patients with each subtype in proportion to that typically seen at the participating institutions.

Though we do not expect an overrepresentation of patients with HER2 positive or TNBC, we will allow a maximum of 15 patients with either of these two subtypes. For the HR positive cohort, we will accrue 10 HR+ patients with an Oncotype DX score >= 25. We will test at most 50 HR+ patients to obtain these 10 HR+ patients with Oncotype DX score >=25.

The pCR rate with 95% confidence interval will be calculated at the end of the study. Patients who received at least 1 cycle of chemotherapy will be evaluable. PFS will be evaluated using Kaplan-Meier methods. pCR rates will be calculated with 95% confidence intervals.

Knowing that some patients may receive tailored treatment with neoadjuvant hormonal treatment and some may not be evaluable, the study will aim to enrol 40 patients evaluable for response. HR positive patients with Oncotype DX recurrence score < 25 will be in an exploratory cohort that will not be included in the primary statistical analysis of the study.

The evaluable intent-to-treat (ITT) population includes all patients with enrolled HER2 positive, triple negative and breast cancers with an Oncotype DX Rescurrence score > 25 that receive at least one cycle of the neoadjuvant regimen.

The population includes all patients with breast cancer who received at least one cycle of the neoadjuvant regimen and had at least one post-infusion tumour assessment.

Overall survival will be calculated from the date of study enrolment to the date of death; for patients who are alive, censoring will be on the date of last contact or date of closing the study, whichever occurs first.

Up to 50 patients with HR positive tumours will be screened to identify at least 10 patients with Oncotype DX recurrence score > 25. The justification for the number of Oncotype DX assays is based upon an estimation that roughly 25% of study patients may be expected to have RS >25. This is based upon results from the following studies:

- Masuda et al., Japanese neoadjuvant study of patients with ER+ T2-3, N0-2 disease: In this study 17/64 (27%) had RS >25[36].
- Gluz et al., West German Plan B trial of an ER+ cohort with T>2 cm, N0 & N+ disease: In this study, 22% had RS >25[37].

Assuming 25% have RS >25, a provision of 40 assays results in a 40% chance of enrolling 9 or fewer patients with RS >25, and a 30% chance of enrolling 8 or fewer patients. However, a provision of 50 assays results in a 16% chance of enrolling 9 or fewer patients with RS >25, and a 9% chance of enrolling 8 or fewer patients. Thus, 50 assays will allow a very good chance of achieving at least 10 patients eligible for enrolment.

7.2Translational Endpoints:

7.2.1NQO1*2 genotype (P187S) status

NQO1*2 genotype (P187S) status will be assessed in all participating subjects. A blood sample or a buccal swab can be used. DNA will be extracted from whole blood or buccal swabs using Qiagen QIAamp minicolumns. The rs1800566 snp will be analysed to determine the NQO1 genotype using an AB7900 real-time thermal cycler using custom probes and primers designed by Applied Biosystems The results will not be used in therapy selection decision-making. At the end of the study response will be correlated to the NQO1*2 genotype (P187S) status results.

7.2.2Pre- and Post Chemotherapy tissue Characteristics

Pre-chemotherapy tissue samples (from biopsy) will be analysed and compared to tissue obtained from breast surgery to characterize immunohistochemical biomarkers between pre- and post-chemotherapy including hormone receptors, HER2 and Ki-67.

7.2.3Optional translational research:

Women participating in this clinical trial will be invited in a separate PICF to donate tissue samples from the pre-chemotherapy biopsy and also tissue obtained from breast surgery after completion of PST.

Women giving informed consent for this part of the study will agree to have their tissue obtained through the clinical study to be used to generate translational models suitable for cancer biology research.

The translational work will involve isolating tumour initiating cells or the so called cancer stem cells from the fresh tissue samples. Also fresh tumour samples will be serially passaged as xenografts in immunocompromised mice without *in vitro* culture to preserve the characteristics of the original tumour. This will be attempted in both the pre-chemotherapy samples and also in the samples obtained after chemotherapy during definite surgical resection.

The collaborating group at Deakin pioneered the approach of using aptamer (chemical antibody) to target surface molecules on cancer stem cells. Experiments will be conducted by treating the xenograft tumours with novel cancer stem cell-targeted and aptamer-directed nanomedicine to assess the translational potential of the novel cancer therapy in targeting breast cancer stem cells.

In addition, previous work that examined the control of histone acetylation on the biology of tumours will be extended to breast cancer. Experiments will be performed examining the hypothesis that epigenetic modification of CSC, through the acetylation of key regulators will alter the CSC quiescent stage and induce cancer cells sensitivity to chemotherapy and radiotherapy.

8Details of the Translational Experiments

8.1Establishing primary breast cancer stem cell xenograft models.

NOD/SCID mice will be maintained under sterile conditions and receive food and water *ad libum*. Nulliparous female mice aged 6 to 8 weeks will be utilized in all experiments. Breast cancer stem cells will be isolated via FACS using cell surface parkers of $EpCAM^+/CD44v6^+/CD24^-$. Approximately 500-800 $EpCAM^+/$ $CD44v6^+/$ $CD24^-$ cells in 200 ul

Matrigel will be injected into the fourth inguinal mammary gland. Tumour formation will be assessed by palpation at least once a week. The breast cancer stem cells will be studied using the following methods:

i. Limiting dilution orthotopic injections used to evaluate tumour initiation,
ii. Serial colony-forming unit, reconstitution and tumorsphere assays will be performed to assess self-renewal and differentiation.
iii. Pulse-chase bromodeoxyuridine (5-bromo-2-deoxyuridine [BrdU]) labeling will be used to examine cell cycle and label-retention of cancer stem cells.
iv. Cells will be treated with paclitaxol and 5-fluorouracil to test selective resistance to chemotherapy, and gene expression profile after chemotherapy will be examined.

Ethical implications for research that involves animals

We will set clear endpoints to ensure that the animal/s welfare is not compromised. Comprehensive project-specific monitoring sheets will be used in this project to ensure the animal welfare. The procedures will be performed on animals and their animal welfare implications are listed below:

- Weighing of animals—placing the animal on scale for a few seconds. This is part of daily handing with little discomfort.
- Implantation of tumour in mammary fat pad—injecting tumour cells into abdominal fat pad. There will be some discomfort with the implanted tumours, but there will be no disruption of the free movement of the animal or eating/drinking.
- Measuring tumour size—using a digital calliper. Brief restraining of the animal. No gross discomfort.
- Tail vein injection—inject small amount of solution into the tail vein. Brief restraining of the animal and discomfort from needle insertion. The discomfort will be short .
- Imaging. Animal will be anaesthetised first and place in imaging instrument for a few minutes. Short discomfort from brief restraining and needle insertion.
- Humane killing. Very short discomfort from needle insertion.

8.2Development of a novel cancer stem cell-targeting nanoparticle

The research group at Deakin University has developed a novel nanoparticle that is able to seek out cancer stem cells and enter the cancer cells to release the anticancer agent as well as burst the cancer cells upon laser irradiation. The key features of the system are:

1) A "GPS" cruise system made of cancer stem cell-targeting EpCAM and CD44v6 RNA chemical antibodies (aptamer) that seeks and reaches to the root of cancer;

2) A multifunctional nanoparticle that combines imaging (for localising tumours), chemotherapy and photothermal therapy will be guided by the smart cancer-seeking GPS (aptamers);

3) The nanoparticle will carry a chemotherapy agents linked by a pH-sensitive bond. Before entering cancer cells, the chemotherapy agents are securely attached to the nanocarrier.

4) Upon reaching cancer stem cells, the nanoparticle will bind to the cancer cell surface marker protein and enter the cancer cells via endocytosis. The imaging component will allow precision imaging using near infrared light; while the chemotherapy drug will be released inside the cancer cells due to the lower pH in the cancer cells' endosome or lysosome.

5) The additional killing power (heating and micro-bubbling) using an entirely different physical mechanism can be unleashed using FDA-approved class III laser at the near-infrared wavelength

6) Extreme limiting dilution assays will be employed to study the efficacy of the novel therapy in the killing of cancer stem cells in in vitro tumour sphere formation and in vivo xenograft transplantation.

9Clinical endpoints

9.1Evaluation of Clinical Activity

9.2Endpoints and assessments:

The study will assess overall objective response rate in the breast at time of definite breast surgery, either breast conserving surgery of mastectomy. Complete (pCR) response defined as breast only, ypT0/ ypTis regardless of nodal status (Figure 1).

9.2.1Histopathology assessment

ER/ PgR and HER2 will be determined on pretreatment biopsy by immunohistochemistry. Hormone receptor (HR) status will be considered positive if ≥10% of tumor cells stained for ER and/or PgR. HER2 status will be assessed by IHC. Samples will subsequently be scored as follows: score 0, membrane staining in ≤10% of tumor cells; score 1+, partial and/or faint membrane staining in >10% of tumor cells; score 2+, weak to moderate, complete membrane staining in >10% tumor cells; and score 3+, strong, complete membrane staining in >10% of tumor cells. ISH will be carried out on all tumors. Tumors with a score of 3+ by IHC or gene amplification by ISH will be considered as HER2 positive.

9.2.2Assessment of clinical tumor response

Tumor diameters will be assessed by physical examination before each treatment cycle and on imaging taken after completion of chemotherapy. Patients will be assessable for tumor response if they received the planned cycles of systemic therapy.

Breast MRI has been shown to be especially useful in the evaluation of response to neoadjuvant therapy. Particular patterns of reduction in tumor size on MRI can be predictive of the likelihood of a successful response. MRI is also capable of detecting residual tumor that is not apparent on mammography or ultrasound. It can also give an accurate evaluation of tumor volume. MRI is already an accepted part of clinical patient evaluation in the neoadjuvant setting in many centers internationally.

Conventional breast imaging with mammography and ultrasound will be performed in all patients with placing a marker in the tumor prior to therapy using mammography or ultrasound as a guide. *Two markers will be placed on each side of the tumor area.*

Mammography or ultrasound may not be feasible in the following categories:
- Women with dense breasts where the tumor is obscured or mammographically occult.
- Young women (age < 30 years) where mammography may not be appropriate.
- Large breasted women where ultrasound may be technically challenging.

Breast MRI will be performed in all 40 evaluable patients before and at the end of chemotherapy treatment.

Residual disease after neoadjuvant treatment includes a broad range of actual responses from near pCR to frank resistance[38]. Patients with a complete absence of invasive tumor cells (irrespective of carcinoma *in situ*) in the surgical specimen of the breast and of the lymph nodes are considered to have a pCR. The response of the breast only is defined as following: A pCR of the breast only. If only scattered tumor cells left it is considered as near complete responders (nCR).

9.2.3Central imaging review

Each patient will have the imaging (U/S, mammogram and/or MRI) undertaken prior and after

completion of chemotherapy locally. MRI assessment of target lesions will also be assessed centrally by radiologists at the main site in Geelong. Findings and interpretations can be discussed between the site and central radiologists.

9.2.4 Surgery

Sentinel node biopsy or axillary dissection at the time of surgery will be at the discretion of the surgeon.

9.3Primary Endpoint:

Overall objective

Response rate in the breast

pCR is defined as no histologic evidence of invasive tumor cells in the surgical breast specimen, with residual DCIS in the breast permitted

Complete (pCR) response defined as breast only, ypT0/ ypTis regardless of nodal status

Pathologic response	Description
ypT0 ypN0	No invasive or non-invasive in breast or nodes
ypT0/is ypN0	No invasive residual in breast or nodes, non-invasive breast residuals allowed
ypT0/is ypN0/+	No invasive residual in breast; non-invasive breast residuals and infiltrated lymph nodes allowed

9.3.1 Histopathological sampling and review

Tumour samples will be examined by a designated pathologist at each site. Clips should be placed around/in the tumour prior to neoadjuvant chemotherapy. Histopathological sampling should be undertaken around clips. If patient consents, any residual invasive cancer tissue will be utilised for translational research.

9.4Secondary end points:

- Response rate in breast and axillary lymph nodes; pCR in breast and axillary lymph nodes (pCR breast and axilla) indicate that there is no evidence of invasive tumor cells in the surgical breast specimen and the axillary nodes.
- Rate of pCR and nCR in the breast combined
- Rate of breast-conserving surgery.
- Safety profile. Toxicity will be assessed using the most current version of the National Cancer Institute Common Terminology Criteria for Adverse Events (NCI-CTCAE) will be used for this assessment. Patients will be evaluable for toxicity if they received at least 1 cycle of chemotherapy, and for efficacy) whether they were eligible and received at least 2 cycles of chemotherapy (evaluable population).

  A Data Safety Monitoring Board (DSMB) will be formed after the first 10 patients are enrolled to independently assess safety of the study treatment and procedures.

- PFS as measured using the Kaplan-Meier method.

10Schedule of assessments

10.1Pre-treatment

The following assessments will be performed within 7 days prior to start of treatment with PST except where noted otherwise:

- Clinical evaluations (including complete physical examination, vital signs including blood pressure and ECOG performance status)
- NQO1*2 genotype (P187S) status. The sample could be either a buccal swab or 4ml EDTA blood.
- Hematology: Full blood count for hemoglobin, platelets and neutrophils within 14 days
- Biochemistry: Serum electrolytes for creatinine and calculation of creatinine clearance. Liver function tests (LFTs) for albumin, alanine transaminase (ALT) or aspartate transaminase (AST), alkaline phosphatase (ALP), and bilirubin within 14 days
- Vitamin D3 level; serum 25(OH) D level.
- 12-lead ECG within 21 days
- Pregnancy test (serum), for women of childbearing potential (defined as not had a hysterectomy or bilateral oophorectomy or not been naturally postmenopausal for at least 24 consecutive months).
- Mammography
- Ultrasonography of the breast and axillary lymph nodes
- CT scan of the chest, abdomen and pelvis and whole body bone scan (or PET scan) within 21 days of commencing study therapy (but ideally as close as possible to start of treatment).
- Whole body bone scintigraphy (Bone Scan) or positron emission tomography/computed tomography (PET/CT).
- Clip placement if clinically indicated according to study clip placement guidelines
- MRI of the breast and axillary nodes in the ITT population

10.2Evaluations during treatment

Treatment will continue routinely unless there is evidence of progressive disease, unacceptable toxicity or patient or investigator request. Assessments will involve the following tests. Please refer to the specific Table for the assessment time-points (Table 1 attached).

- History and assessment of disease related symptoms
- Physical examination
- Toxicity and adverse event assessment
- Clinical evaluations (including vital signs and ECOG performance status)
- Hematology: Full blood count for hemoglobin, platelets and neutrophils
- Biochemistry: Serum electrolytes for creatinine. Liver function tests (LFTs) for albumin, alanine transaminase (ALT) or aspartate transaminase (AST), alkaline phosphatase (ALP) and bilirubin.
- Imaging: Ultrasonography of the breast and axillary lymph nodes and mammography about 2 weeks prior to the end of PST.
- MRI of the breast axillary nodes in the ITT population
- No other study-specific radiological assessments are required for the study, but may be performed as clinically indicated.

10.3Evaluations after treatment

After completion of study therapy, patients should be reviewed 2 weeks after the end of PST and 2-4 weeks after definitive surgery. Further therapy and investigations are at the investigator’s discretion. All patients will be followed for survival status every 12 months until death, lost to follow-up, or withdrawal of consent to survival follow-up for period of 5 years.

Table 1 – Timing of Study Procedures

Study Time-Point	Pre-Treatment (7 days prior to start of PST unless otherwise indicated)	Cycle 1 (EC)[1]	Cycle 2 (EC)[1]	Cycle 3 (EC)[1]	Cycle 4 (EC)[1]	Cycle 5 NABP[2]	Cycle 6 NABP[2]	Cycle 7 NABP[2]	2 weeks After the end of PST	Surgery	2-4 week after definite surgery	Survival phase
Informed consent	x (-28 days)											
Medical History including familial breast, ovarian and other cancer history. Prior anti-neoplastic therapy	x	X	x	x	x	x	x	x	x		X	
Physical examination	x	X	x	x	x	x	x	x	x		X	
Vital signs (including BP)	x	X	x	x	x	x	x	x	x			
ECOG performance status	x	X	x	x	x	x	x	x	x			
NQO1*2 genotype (P187S) status via either buccal swab or 4 ml EDTA blood	x											
Familial history of cancer	x											
Diagnosis of cancer (ER, PR and HER2 status including Ki67 index	x											
Oncotype DX in HR positive patients	x											
Translational research pre-chemo core biopsy[3]	x											
Haematology – full blood count for haemoglobin, platelets, neutrophils	X (-14 days)	X	x	x	x	x	x	x	x			
Biochemistry – serum for creatinine clearance	X (-14 days)	X	x	x	x	x	x	x	x			
Liver function tests: albumin, ALT, ALP, bilirubin. AST if LFTs are abnormal	X (-14 days)	X	x	x	x	x	x	x	x			
Vitamin D3 level, Serum 25 (OH) D level	x											
12-lead ECG	x (-21 days)											
Left ventricular ejection fraction by ECHO/ MUGA	X (-3 months)											
Serum pregnancy test[4]	X											

**Study Time-Point**	**Pre-Treatment (7 days prior to start of PST unless otherwise indicated)**	**Cycle 1 (EC)[1]**	**Cycle 2 (EC)[1]**	**Cycle 3 (EC)[1]**	**Cycle 4 (EC)[1]**	**Cycle 5 NABP[2]**	**Cycle 6 NABP[2]**	**Cycle 7 NABP[2]**	**2 weeks After the end of PST**	**Surgery**	**2-4 week after definite surgery**	**Survival phase**
Mammogram and/ or Ultrasound of breast and axillary lymph nodes	X (21 days)								X			
MRI of breast and axillary lymph nodes	X								X			
Clip placement and images[5]	X										X	
CT scan of chest, abdomen, pelvis and whole body bone scan (or PET scan)	X (-21 days)											
Translational research post-chemo surgical tissue[3]										X		
Histopathological tumour response evaluation											X	
Survival												X

Legend:

1. Each cycle of EC is of 3 weeks duration
2. NABP is for 12 weeks duration
3. Tissue is only collected from patients who have consented for tissue study
4. Only for women of childbearing potential
5. Clip placement where clinically indicated

10.3.1 Patient related outcome measures

Patients will be followed up after completion of study until death or withdrawal from study for the following reasons:

- Rate of breast-conserving surgery.
- Survival (vital status and date of death).
- Progression and date of progression.

11 Patient safety (Monitoring of Serious Adverse Events)

11.1 Adverse events

An adverse event (AE) is any noxious, unintended, or untoward medical occurrence that may appear or worsen in a subject during the course of a study. It may be a new intercurrent illness, a worsening concomitant illness, an injury, or any concomitant impairment of the subject's health, including laboratory test values (as specified by the criteria below), regardless of aetiology. Any worsening (i.e., any clinically significant adverse change in the frequency or intensity of a pre-existing condition) should be considered an AE. A diagnosis or syndrome should be recorded on the AE page of the case report form (CRF) rather than the individual signs or symptoms of the diagnosis or syndrome.

An overdose, accidental or intentional, whether or not it is associated with an AE, or abuse, withdrawal, sensitivity or toxicity to an investigational product should be reported as an AE. If an overdose is associated with an AE, the overdose and adverse event should be reported as separate terms.

All subjects will be monitored for AEs during the study. Assessments may include monitoring of any or all of the following parameters: the subject's clinical symptoms, laboratory, pathological, radiological or surgical findings, physical examination findings, or other appropriate tests and procedures

All AEs will be recorded by the Investigator from the time the subject signs informed consent to 28 days after the last dose of IP or until the last study visit, whichever period is longer. AEs and serious adverse events (SAEs) will be recorded on the AE page of the CRF and in the subject's source documents. All SAEs must be reported to The Andrew Love Cancer Centre, Clinical Trial Centre, within 24 hours of the Investigator's knowledge of the event by facsimile, or other appropriate method, using the SAE Report Form, or approved equivalent form. The Trial centre will be responsible for forwarding these reports onto Celgene.

Twenty-eight days after the last dose of IP is the minimum time to collect AEs but it may be longer. For example, study-related AEs occurring beyond this time-frame may need to be documented during a long-term follow-up period and can be described in this section of the protocol, if applicable.

Toxicities will be assessed at each visit. Version 4.0 of the National Cancer Institute Common Terminology Criteria for Adverse Events (NCI-CTCAE) will be used for this assessment. Proportions of patients with grade 3 or 4 toxicity will be determined.

11.2 Reporting of Serious Adverse Events (SAE)

A qualified Investigator will evaluate all adverse events as to meeting serious criteria, severity and causality. :

A serious adverse event (toxicity) or reaction is any untoward medical occurrence that at any dose:

- results in death
- is life-threatening (NOTE: The term “life-threatening” in the definition of “serious” refers to an event in which the patient was at risk of death at the time of the event; it does not refer to an event which hypothetically might have caused death if it were more severe.)
- requires in-patient hospitalisation or prolongation of existing hospitalisation
- results in persistent or significant disability/incapacity
- is a congenital anomaly/birth defect, or
- is medically significant, i.e. is considered to be important but does not meet any of the above criteria. Medical and scientific judgment should be exercised in deciding whether reporting an event as an SAE is appropriate in the latter situation. Examples of medically significant events are intensive treatment in an emergency room, or at home for allergic bronchospasm; blood dyscrasias or convulsions that do not result in hospitalisation; development of drug dependency or drug abuse; or malignant tumours when they are histologically different from the primary tumour.

11.3Other events to be treated as SAEs

11.3.1Exposure to drug during pregnancy/lactation

In principle, pregnancy and the lactation period are exclusion criteria. In the event of a pregnancy occurring during the course of a study, the patient must be withdrawn from study drug immediately. Pregnancies occurring up to 6 months after the completion of the test drug must also be reported to the investigator. The investigator should counsel the patient, discuss the risks of continuing with the pregnancy and the possible effects on the foetus.

Parental and neonatal outcomes must be recorded even if they are completely normal and without AEs. The Celgene pregnancy form should be used, even though pregnancy is not considered a SAE. No "serious criterion" should be ticked. The SAE form is used to ensure expedited reporting.

11.4Events not treated as SAEs

Progression or deterioration of disease is not to be regarded as an SAE. This is unlikely but possible in early breast cancer.

Due to the seriousness of the disease in this study, certain conditions defined as SAEs will be excluded from reporting:

- events (including hospitalisations and deaths) which are clearly due to cancer or its progression
- elective hospitalisation and surgery for treatment of disease however, hospitalization or prolonged hospitalization for a complication remains a reportable SAE.
- elective hospitalisation to simplify treatment or study procedures, however hospitalization or prolonged hospitalization for a complication remains a reportable SAE.
- A standard procedure for protocol therapy administration. However, hospitalization or prolonged hospitalization for a complication of therapy administration will be reported as an SAE.
- Routine treatment or monitoring of the studied indication not associated with any deterioration in condition.
- The administration of blood or platelet transfusion as routine treatment of studied indication. However, hospitalization or prolonged hospitalization for a complication of such transfusion remains a reportable SAE.

- A procedure for protocol/disease-related investigations (e.g., surgery, scans, endoscopy, sampling for laboratory tests, bone marrow sampling). However, hospitalization or prolonged hospitalization for a complication of such procedures remains a reportable SAE.
- Hospitalization or prolongation of hospitalization for technical, practical, or social reasons, in absence of an AE.
- Emergency outpatient treatment or observation that does not result in admission, unless fulfilling other seriousness criteria above.
-

11.5Methods of recording and assessing toxicity (Adverse Events)
All AEs must be documented in the toxicity case report form (CRFs). Among these AEs, all SAEs must be additionally documented using a SAE form.

The following aspects must be recorded for each event in the CRF:
- A description of the AE in medical terms according to the definitions in NCI CTCAE, Version 4.0, not as reported by the patient.
- The grade/severity as assessed by the investigator according to the definitions in NCI CTCAE, Version 4.0:
    - Grade 1 = mild
    - Grade 2 = moderate
    - Grade 3 = severe and undesirable
    - Grade 4 = life-threatening or disabling
- Seriousness: yes or no

If in any one patient the same AE occurs on several occasions, then the AE in question must be documented and assessed as new each time.

11.6Recording and assessing SAEs
The following aspects must be recorded in the SAE form:
- A description of the SAE in medical terms, not as reported by the patient; only the term(s) that fulfils the seriousness criteria should be listed.
- The date of onset (start date).
- The date of recovery (stop date).
- The grade/severity as assessed by the investigator according to the definitions in NCI CTCAE, Version 4.0:
    - Grade 1 = mild
    - Grade 2 = moderate
    - Grade 3 = severe,
    - Grade 4 = life-threatening
    - Grade 5 = death
- The causal relationship to all lithium as assessed by the investigator; the decisive factor in the documentation is the temporal relation between the AE and the study drug. The following judgments of the causality to study drug or study procedures are to be used:
    - 1 = related to study drug
    - 2 = unrelated to study drug
- Whether the SAE is consistent with the expected side-effect profile of the study agents, as judged by the investigator and with reference to the current investigator brochures and product information. Common expected SAEs are listed in the Appendix. SAEs which are consistent with the known side-effect profile, but which are associated with an unexpected outcome should be classified as unexpected.
- The outcome according to the following definitions:
    - 1 = recovered
    - 2 = recovered with sequelae, describe sequelae: ________________________.
    - 3 = ongoing
    - 4 = death

- 5 = other, specify: ____

Seriousness, not severity, serves as a guide for defining regulatory obligations.

11.7 Causality

The Investigator must determine the relationship between the administration of IP and the occurrence of an AE/SAE as Not Suspected or Suspected as defined below:

Not suspected:	The temporal relationship of the adverse event to IP administration makes **a causal relationship unlikely or remote**, or other medications, therapeutic interventions, or underlying conditions provide a sufficient explanation for the observed event.
Suspected:	The temporal relationship of the adverse event to IP administration makes **a causal relationship possible**, and other medications, therapeutic interventions, or underlying conditions do not provide a sufficient explanation for the observed event.

11.8 Duration

For both AEs and SAEs, the Investigator will provide a record of the start and stop dates of the event.

11.9 Action Taken

The Investigator will report the action taken with IP as a result of an AE or SAE, as applicable (e.g., discontinuation or reduction of IP, as appropriate) and report if concomitant and/or additional treatments were given for the event.

11.10 Outcome

The investigator will report the outcome of the event for both AEs and SAEs. All SAEs that have not resolved upon discontinuation of the subject's participation in the study must be followed until recovered, recovered with sequelae, not recovered (death due to another cause) or death (due to the SAE).

11.11 Abnormal Laboratory Values

An abnormal laboratory value is considered to be an AE if the abnormality:

- results in discontinuation from the study;
- requires treatment, modification/ interruption of IP dose, or any other therapeutic intervention; or
- Is judged to be of significant clinical importance.

Regardless of severity grade, only laboratory abnormalities that fulfil a seriousness criterion need to be documented as a serious adverse event.

If a laboratory abnormality is one component of a diagnosis or syndrome, then only the diagnosis or syndrome should be recorded on the AE page/screen of the CRF. If the abnormality was not a part of a diagnosis or syndrome, then the laboratory abnormality should be recorded as the AE.

11.12 Females of Childbearing Potential:

Pregnancies and suspected pregnancies (including a positive pregnancy test regardless of age

or disease state) of a female subject occurring while the subject is on IP, or within 28 days of the subject’s last dose of IP, are considered immediately reportable events. IP is to be discontinued immediately and the subject instructed to return any unused portion of the IP to the Investigator. The pregnancy, suspected pregnancy, or positive pregnancy test must be reported to the Haematology and Oncology Trials Team who will inform Celgene immediately using the Pregnancy Reporting Form provided by Celgene or an approved equivalent form].

The female subject may be referred to an obstetrician-gynaecologist (not necessarily one with reproductive toxicity experience) or another appropriate healthcare professional for further evaluation.

The Investigator will follow the female subject until completion of the pregnancy, and must notify Haematology and Oncology Trials Team immediately about the outcome of the pregnancy (either normal or abnormal outcome).

If the outcome of the pregnancy was abnormal (e.g., spontaneous or therapeutic abortion), the Investigator should report the abnormal outcome as an AE. If the abnormal outcome meets any of the serious criteria, it must be reported as an SAE within 24 hours of the Investigator’s knowledge of the event using the SAE Report Form, or approved equivalent form.

All neonatal deaths that occur within 28 days of birth should be reported, without regard to causality, as SAEs. In addition, any infant death after 28 days that the Investigator suspects is related to the in utero exposure to the IP should also be reported within 24 hours of the Investigator’s knowledge of the event using the SAE Report Form, or approved equivalent form.

11.13Expedited Reporting of Adverse Events

11.13.1Reporting to Regulatory Authorities and the Ethics Committee

The Haematology and Oncology Trials Team will inform relevant Regulatory Authorities and Ethics Committees;

-Of all relevant information about serious unexpected adverse events suspected to be related to the IP that are fatal or life-threatening as soon as possible, and in any case no later than seven days after knowledge of such a case. Relevant follow-up information for these cases will be subsequently be submitted within an additional eight days

-Of all other serious unexpected events suspected to be related to the IP as soon as possible, but within a maximum of fifteen days of first knowledge by the investigator.

11.14Immediate reporting by Investigator to the Haematology and Oncology Trials Team and the Haematology and Oncology Trials Team to Celgene

The investigator will inform the the Haematology and Oncology Trials Team of all SAEs within 24 hours in order that the The Haematology and Oncology Trials Team can fulfill their regulatory reporting obligations within the required timeframes.

The Haematology and Oncology Trials Team will supply Celgene with a copy of all SAEs which involve *exposure* to a Celgene product within 24 hours of being made aware of the event regardless of whether or not the event is listed in the reference document (e.g. IB, SmPC).

The Haematology and Oncology Trials Team will provide Celgene with a copy of the annual periodic safety report e.g. Development Update Safety Report (DSUR) at the time of submission to the Regulatory Authority and Ethics Committee.

11.15Safety instructions specific to the study

Toxicity will be recorded for all patients (including those withdrawing from the study treatment because of toxicity) for 28 days following the last dose of study drug. Adverse events related to study drug(s) that are observed, either during study treatment, or prior to the thirtieth day following the last dose of study drug(s), will be followed until resolution or stabilisation.

12Ethical principles

The study will be performed in accordance with the ICH GCP Guidelines.

12.1 Informed consent

Participants will be given a full explanation, in lay terms, of the aims of the study and the risks involved. It will be explained that they may refuse to take part in, or withdraw from the study without prejudice to their future care and treatment. Written informed consent will be obtained from all participants. Consent will be obtained both verbally and in writing after the participant has read the Participant Information Sheet. Sample versions of the Participant Information Sheet and Consent Form are appended.

12.2 Confidentiality

All data generated in this study will remain confidential and securely stored and no report will contain any reference to participants' names. If data is transferred inter or intra-state information will be transported in a coded fashion and participant names will not be included.

12.3 Study Monitoring

12.4 Responsibilities of the investigators

The Investigator(s) undertake(s) to perform the study in accordance with ICH Good Clinical Practice Guidelines.

The Investigator is required to ensure compliance with respect to the investigational drug schedule, visit schedule and procedures required by the protocol. The Investigator agrees to provide all information requested in the Case Report Form in an accurate manner according to the instructions provided.

12.5 Data collection and quality assurance

12.5.1Data Collection Forms and database

The Trial Centre will send case report forms to individual sites for completion. Originals of completed forms should be sent to the Trial Centre regularly after completion with photocopies being retained at the enrolling institution.

Additionally to facilitate analysis data should be submitted into the Neonab database via KeyTrust account access to the Barwon Health intranet, the appropriate investigator-designated research staff will log in with the pre-assigned user names and passwords. Each web section is separated into modules; each module must be saved. The user selects the link to the appropriate form and enters data directly into the web-based form. Forms can be completed in more than one sitting as long as the user saves after each section and completed at a later date.
In the event of technical problems preventing access to the Data Centre website, site coordinator should notify the Trial Centre.

12.5.2 Monitoring

Data will be monitored to assess compliance with the protocol and to look for unforeseen trends that may be indicative of procedural differences among clinical sites. If patterns are discovered in the data that appear to arise from causes specific to an institution, the Trial Centre will contact the site to resolve the problem. The Trial Centre will conduct eligibility checks for all patients throughout the study primarily via the electronic database; copies of relevant documents (such as signed consent forms, pathology reports and radiology reports) may be requested and monitored by the Trial Centre if necessary for CRF edit checks and source data verification. All records including medical histories, radiological imaging and laboratory tests must be considered 'source data' and retained for at least 15 years after completion of the trial and remain available for audit if required in accordance with Good Clinical Practice. Source documents must be maintained for this study in order to ensure compliance with GCP. Source documents and CRFs may be reviewed by the local PI.

12.5.3 Protocol Deviations and violations

A protocol deviation is defined as any departure from what is described in the protocol. Specifically, it applies to deviations related to:

- -Randomisation of ineligible participant (s)
- -Eligibility criteria breach
- -Screening procedure required by the protocol not done
- -Screening or on- project procedure done outside the protocol required time
- -Incorrect therapy given to participant(s)
- -On- project procedure required by the protocol not completed as determined by Principal Investigator
- -Visit non- compliance
- -Medication non- compliance

Deviations with no effects on the risk/benefit ratio of the clinical trial (such as minimal delays in assessments or visits) will be distinguished from those above that might have an effect on this risk/benefit ratio.

12.6 The use and completion of case report forms (CRFs)

It is the responsibility of the Investigator to prepare and maintain adequate and accurate CRF's, recording all observations and other data pertinent to the clinical investigation in a timely manner.

12.7 Record retention in investigator centres

The Investigator must maintain all study records, patient files and other source data for the maximum period of time permitted by the hospital, institution or private practice. The Investigator is required to arrange for the retention of the patient identification codes and records for at least 15 years after the completion or discontinuation of the trial.

12.8 Publications

Results will be analysed and presented at national and international conferences. Subsequently at least one peer reviewed publication will follow with authorship decided depending on contribution of investigators to the study.

12.9 Protocol amendments

Any change agreed upon will be recorded in writing, the written amendment will be circulated to the Investigator and the amendment will be appended to this protocol. The HREC should be informed of any significant deviations.

If the change or deviation increase risk to the study population, or adversely affects the validity of the clinical investigation or the patient's rights, full approval/advice must be obtained prior to implementation. For changes that do not involve increased risk or affect the validity of the investigation or the patient's rights, approval/advice may be obtained by expedited review, where applicable.

In some instances, an amendment may require a change to a consent form. The Investigator must receive approval/advice of the revised consent form prior to implementation of the change.

13References:

1. Foulkes, W.D., I.E. Smith, and J.S. Reis-Filho, *Triple-negative breast cancer.* N Engl J Med, 2010. **363**(20): p. 1938-48.
2. Kaufmann, M., et al., *Recommendations from an international expert panel on the use of neoadjuvant (primary) systemic treatment of operable breast cancer: an update.* Journal of Clinical Oncology, 2006. **24**(12): p. 1940.
3. Bear, H.D., et al., *The effect on tumor response of adding sequential preoperative docetaxel to preoperative doxorubicin and cyclophosphamide: preliminary results from National Surgical Adjuvant Breast and Bowel Project Protocol B-27.* Journal of clinical oncology : official journal of the American Society of Clinical Oncology, 2003. **21**(22): p. 4165-74.
4. Gradishar, W.J., et al., *Neoadjuvant docetaxel followed by adjuvant doxorubicin and cyclophosphamide in patients with stage III breast cancer.* Annals of Oncology, 2005. **16**(8): p. 1297-1304.
5. von Minckwitz, G., et al., *Definition and Impact of Pathologic Complete Response on Prognosis After Neoadjuvant Chemotherapy in Various Intrinsic Breast Cancer Subtypes.* Journal of Clinical Oncology, 2012. **30**(15): p. 1796-1804.
6. Foote, M., *Using nanotechnology to improve the characteristics of antineoplastic drugs: improved characteristics of nab-paclitaxel compared with solvent-based paclitaxel.* Biotechnology annual review, 2007. **13**: p. 345-57.
7. Gradishar, W.J., et al., *Significantly longer progression-free survival with nab-paclitaxel compared with docetaxel as first-line therapy for metastatic breast cancer.* Journal of clinical oncology : official journal of the American Society of Clinical Oncology, 2009. **27**(22): p. 3611-9.
8. Gradishar, W.J., et al., *Phase III trial of nanoparticle albumin-bound paclitaxel compared with polyethylated castor oil-based paclitaxel in women with breast cancer.* Journal of clinical oncology : official journal of the American Society of Clinical Oncology, 2005. **23**(31): p. 7794-803.
9. Yardley, D., et al., *A pilot study of adjuvant nanoparticle albumin-bound (nab) paclitaxel and cyclophosphamide, with trastuzumab in HER2-positive patients, in the treatment of early-stage breast cancer.* Breast cancer research and treatment, 2010. **123**(2): p. 471-5.
10. Dang, C., et al., *The safety of dose-dense doxorubicin and cyclophosphamide followed by paclitaxel with trastuzumab in HER-2/neu overexpressed/amplified breast cancer.* Journal of clinical oncology : official journal of the American Society of Clinical Oncology, 2008. **26**(8): p. 1216-22.
11. Kaklamani, V.G., et al., *Pilot neoadjuvant trial in HER2 positive breast cancer with combination of nab-paclitaxel and lapatinib.* Breast cancer research and treatment, 2011.
12. Khasraw, M., E. Brogi, and A.D. Seidman, *The need to examine metastatic tissue at the time of progression of breast cancer: is re-biopsy a necessity or a luxury?* Current oncology reports, 2011. **13**(1): p. 17-25.
13. Kumaki, N., et al., *Alteration of immunohistochemical biomarkers between pre- and post-chemotherapy: hormone receptors, HER2 and Ki-67.* Breast cancer, 2011.
14. Iwamoto, T., et al., *Gene pathways associated with prognosis and chemotherapy sensitivity in molecular subtypes of breast cancer.* Journal of the National Cancer Institute, 2011. **103**(3): p. 264-72.
15. Partridge, S.C., et al., *MRI measurements of breast tumor volume predict response to neoadjuvant chemotherapy and recurrence-free survival.* American Journal of Roentgenology, 2005. **184**(6): p. 1774-1781.
16. O'Flynn, E.A. and N.M. Desouza, *Functional magnetic resonance: biomarkers of response in breast cancer.* Breast cancer research : BCR, 2011. **13**(1): p. 204.
17. Robidoux, A., et al., *A phase II neoadjuvant trial of sequential nanoparticle albumin-bound paclitaxel followed by 5-fluorouracil/epirubicin/cyclophosphamide in locally advanced breast cancer.* Clinical breast cancer, 2010. **10**(1): p. 81-6.
18. Findlay, B.P., et al. *Epirubicin, as a Single Agent or in Combination, for Metastatic Breast Cancer Practice Guideline Report # 1-6.* The Practice Guidelines Initiative is sponsored by: Cancer Care Ontario & the Ontario Ministry of Health and Long-term

Care. 2003; Available from: http://www.cancercare.on.ca/common/pages/UserFile.aspx?serverId=6&path=/File%20Database/CCO%20Files/PEBC/pebc1-6f.pdf.

19. Findlay, B., et al., *A dose escalation trial of adjuvant cyclophosphamide and epirubicin in combination with 5-fluorouracil using G-CSF support for premenopausal women with breast cancer involving four or more positive nodes.* Annals of oncology, 2007. **18**(10): p. 1646.
20. Khasraw, M., R. Bell, and C. Dang, *Epirubicin: Is it like doxorubicin in breast cancer? A clinical review.* The Breast, 2012.
21. Goldhirsch, A., et al., *Strategies for subtypes—dealing with the diversity of breast cancer: highlights of the St Gallen International Expert Consensus on the Primary Therapy of Early Breast Cancer 2011.* Annals of oncology, 2011. **22**(8): p. 1736-1747.
22. Dowsett, M., et al., *Assessment of Ki67 in breast cancer: recommendations from the International Ki67 in Breast Cancer Working Group.* Journal of the National Cancer Institute, 2011. **103**(22): p. 1656-1664.
23. Untch, M., et al., *Neoadjuvant Clinical Trials for the Treatment of Primary Breast Cancer: The Experience of the German Study Groups.* Current oncology reports, 2012: p. 1-8.
24. Perou, C.M., et al., *Molecular portraits of human breast tumours.* Nature, 2000. **406**(6797): p. 747-752.
25. Cheang, M.C.U., et al., *Ki67 Index, HER2 Status, and Prognosis of Patients With Luminal B Breast Cancer.* Journal of the National Cancer Institute, 2009. **101**(10): p. 736-750.
26. Paik, S., et al., *Gene expression and benefit of chemotherapy in women with node-negative, estrogen receptor,Äìpositive breast cancer.* Journal of Clinical Oncology, 2006. **24**(23): p. 3726-3734.
27. al, V.e., *Cells of origin in cancer.* Nature, 2011. **469**(7330): p. 314-322.
28. Zoller, M., *CD44: can a cancer-initiating cell profit from an abundantly expressed molecule?* Nature Reviews Cancer, 2011. **11**(4): p. 254-267.
29. Al-Hajj, M., et al., *Prospective identification of tumorigenic breast cancer cells.* Proceedings of the National Academy of Sciences, 2003. **100**(7): p. 3983-3988.
30. Stratford, A.L., et al., *Targeting tumour-initiating cells to improve the cure rates for triple-negative breast cancer.* Expert reviews in molecular medicine, 2010. **12**(1).
31. Hebbard, L., et al., *CD44 expression and regulation during mammary gland development and function.* Journal of Cell Science, 2000. **113**(14): p. 2619-2630.
32. Munz, M., P.A. Baeuerle, and O. Gires, *The emerging role of EpCAM in cancer and stem cell signaling.* Cancer Research, 2009. **69**(14): p. 5627.
33. Shigdar, S., et al., *Clinical applications of aptamers and nucleic acid therapeutics in haematological malignancies.* British journal of haematology, 2011.
34. Yu, C., et al., *Novel aptamer-nanoparticle bioconjugates enhances delivery of anticancer drug to MUC1-positive cancer cells in vitro.* PloS one, 2011. **6**(9): p. e24077.
35. Wu, S., et al., *Aptamer-functionalized magnetic nanoparticle-based bioassay for the detection of ochratoxin A using upconversion nanoparticles as labels.* The Analyst, 2011. **136**(11): p. 2306-14.
36. Masuda, N., et al., *A study of the recurrence score by the 21-gene signature assay as a predictor of clinical response to neoadjuvant exemestane for 24 weeks in estrogen-receptor-positive breast cancer.* ASCO Meeting Abstracts, 2011. **29**(15_suppl): p. 558.
37. Gluz, O., et al., *Prospective comparison of recurrence score and independent central pathology assessment of prognostic tools in early breast cancer (BC): Focus on HER2, ER, PR, Ki-67 results from the phase III WSG-Plan B trial.* ASCO Meeting Abstracts, 2012. **30**(15_suppl): p. 552.
38. Symmans, W.F., et al., *Measurement of Residual Breast Cancer Burden to Predict Survival After Neoadjuvant Chemotherapy.* Journal of Clinical Oncology, 2007. **25**(28): p. 4414-4422.
